# International consensus recommendations on the diagnostic work-up for malformations of cortical development

**DOI:** 10.1038/s41582-020-0395-6

**Published:** 2020-09-07

**Authors:** Renske Oegema, Tahsin Stefan Barakat, Martina Wilke, Katrien Stouffs, Dina Amrom, Eleonora Aronica, Nadia Bahi-Buisson, Valerio Conti, Andrew E. Fry, Tobias Geis, David Gomez Andres, Elena Parrini, Ivana Pogledic, Edith Said, Doriette Soler, Luis M. Valor, Maha S. Zaki, Ghayda Mirzaa, William B. Dobyns, Orly Reiner, Renzo Guerrini, Daniela T. Pilz, Ute Hehr, Richard J. Leventer, Anna C. Jansen, Grazia M. S. Mancini, Nataliya Di Donato

**Affiliations:** 1 Department of Genetics, University Medical Center Utrecht, Utrecht University, Utrecht, Netherlands; 2 Department of Clinical Genetics, Erasmus MC University Medical Center, Rotterdam, Netherlands; 3 Centre for Medical Genetics, UZ Brussel, Reproduction and Genetics, Vrije Universiteit Brussel, Brussels, Belgium; 4 Pediatric Neurology, Kannerklinik, Centre Hospitalier de Luxembourg, Luxembourg, Grand Duchy of Luxembourg; 5 Pediatric Neurology, Hôpital Universitaire des Enfants Reine Fabiola, Université Libre de Bruxelles, Brussels, Belgium; 6 Amsterdam UMC, University of Amsterdam, Department of (Neuro)pathology, Amsterdam, Netherlands; 7 Stichting Epilepsie Instellingen Nederland (SEIN), Amsterdam, Netherlands; 8 Pediatric Neurology, Necker Enfants Malades, University Hospital Imagine Institute, Paris, France; 9 Pediatric Neurology, Neurogenetics and Neurobiology Unit and Laboratories, Department of Neuroscience, A. Meyer Children’s Hospital, University of Florence, Florence, Italy; 10 Institute of Medical Genetics, University Hospital of Wales, Cardiff, UK; 11 Division of Cancer and Genetics, School of Medicine, Cardiff University, Cardiff, UK; 12 Department of Pediatric Neurology, Klinik St Hedwig, University Children’s Hospital Regensburg (KUNO), Regensburg, Germany; 13 Child Neurology, Hospital Universitari Vall d’Hebron, Barcelona, Spain; 14 Department of Biomedical Imaging and Image Guided Therapy, Medical University of Vienna, Vienna, Austria; 15 Section of Medical Genetics, Mater dei Hospital, Msida, Malta; 16 Department of Anatomy and Cell Biology, University of Malta, Msida, Malta; 17 Department of Paediatrics, Mater dei Hospital, Msida, Malta; 18 Hospital Universitario Puerta del Mar, INiBICA, Puerta, Spain; 19 Clinical Genetics Department, Human Genetics and Genome Research Division, National Research Centre, Cairo, Egypt; 20 Department of Pediatrics, Division of Genetics and Metabolism, University of Minnesota, Minneapolis, MN USA; 21 Department of Molecular Genetics, Weizmann Institute of Science, Rehovot, Israel; 22 West of Scotland Clinical Genetics Service, Queen Elizabeth University Hospital, Glasgow, UK; 23 Center for Human Genetics Regensburg, Regensburg, Germany; 24 Department of Neurology, Royal Children’s Hospital, Murdoch Children’s Research Institute and University of Melbourne Department of Paediatrics, Melbourne, VIC Australia; 25 Pediatric Neurology Unit, Department of Pediatrics, UZ Brussel, Neurogenetics Research Group, Vrije Universiteit Brussel, Brussels, Belgium; 26 ENCORE Expertise Center for Neurodevelopmental Disorders, Erasmus MC University Medical Center, Rotterdam, Netherlands; 27 Institute for Clinical Genetics, TU Dresden, Dresden, Germany

**Keywords:** Neurodevelopmental disorders, Paediatric neurological disorders

## Abstract

Malformations of cortical development (MCDs) are neurodevelopmental disorders that result from abnormal development of the cerebral cortex in utero. MCDs place a substantial burden on affected individuals, their families and societies worldwide, as these individuals can experience lifelong drug-resistant epilepsy, cerebral palsy, feeding difficulties, intellectual disability and other neurological and behavioural anomalies. The diagnostic pathway for MCDs is complex owing to wide variations in presentation and aetiology, thereby hampering timely and adequate management. In this article, the international MCD network Neuro-MIG provides consensus recommendations to aid both expert and non-expert clinicians in the diagnostic work-up of MCDs with the aim of improving patient management worldwide. We reviewed the literature on clinical presentation, aetiology and diagnostic approaches for the main MCD subtypes and collected data on current practices and recommendations from clinicians and diagnostic laboratories within Neuro-MIG. We reached consensus by 42 professionals from 20 countries, using expert discussions and a Delphi consensus process. We present a diagnostic workflow that can be applied to any individual with MCD and a comprehensive list of MCD-related genes with their associated phenotypes. The workflow is designed to maximize the diagnostic yield and increase the number of patients receiving personalized care and counselling on prognosis and recurrence risk.

## Abstract (web summary)

Malformations of cortical development (MCDs) are neurodevelopmental disorders that result from abnormal development of the cerebral cortex in utero. In this Consensus Statement, the international MCD network Neuro-MIG provides recommendations to aid both expert and non-expert clinicians in the diagnostic work-up of MCDs.

## Introduction

Abnormal formation of the cerebral cortex in utero leads to neurodevelopmental disorders known as malformations of cortical development (MCDs). Although individually rare, as a group MCDs represent a major cause of intellectual disability, autism, epilepsy and cerebral palsy^1,2^. The last update of the developmental and genetic classification for MCDs, which was published in 2012, includes 200 clinical entities and classifies them into three major groups: malformations secondary to abnormal neuronal and glial cell proliferation and apoptosis, including microcephaly and macrocephaly; neuronal migration disorders, represented by heterotopia, lissencephaly and cobblestone malformation (COB); and malformations of postmigrational cortical organization and connectivity, represented by conditions such as polymicrogyria, schizencephaly and focal cortical dysplasia (FCD)^3^.

Many MCDs are caused by an underlying genetic defect. Rapid advances in molecular genetics and neuroimaging techniques in recent years have substantially increased the number of recognized MCD forms and their associated genes, and have highlighted the considerable genetic heterogeneity associated with these disorders^1^. Next-generation sequencing (NGS) of a selection of genes related to a phenotype (gene panel), the coding exons of the human genes (exome sequencing) or the genome of an individual (genome sequencing) has enabled rapid sequencing of large numbers of genes.

Even following intensive diagnostic assessments, many individuals with an MCD remain without a molecular diagnosis^4–6^. The complex nature and high degree of clinical and genetic heterogeneity of MCDs demand highly specialized and multidisciplinary expertise. However, MCD experts usually work individually or in small multidisciplinary teams. Currently, comprehensive guidelines for diagnosis and management are lacking, adding to the variability in the diagnostic approach between different centres. The disease course and long-term clinical outcome are often difficult to predict at an early stage, and medical management is rarely evidence-based. These challenges highlight the need for an expert-driven multidisciplinary effort to better understand these disorders. The availability of carefully curated MCD gene panels to the wider medical community will enable accurate molecular diagnosis in a larger number of patients without long delays or unnecessary investigations.

We established the international multidisciplinary network Neuro-MIG with the aim of disseminating knowledge to the broad medical community, improving the diagnosis and management of MCDs and accelerating research into MCDs^7^. In this article, we first review the clinical presentation and aetiology of the main MCD types. On the basis of a critical review of the literature, expert surveys and discussions, we then present a consensus statement on the clinical and molecular investigations in patients with MCDs, including specific recommendations on clinical work-up, molecular diagnostic methods and alternative strategies in undiagnosed patients.

## Methods

This article represents a consensus document based on three face-to-face expert meetings within the Neuro-MIG network that were held in St Julians, Malta, from 21 to 23 February 2018, in Lisbon, Portugal, on 13 and 14 September 2018, and in Rehovot, Israel, on 17 March 2019. The meetings were funded by the European Cooperation in Science & Technology (COST Action CA16118). Two Neuro-MIG working groups, WG1 and WG3, took the lead in preparing the draft, although a larger group within the network was invited to participate in the Delphi consensus procedure and comment on the second draft. The final version of the consensus document was reviewed by the drafting team and circulated among all COST network members before submission.

PubMed was systematically queried for phenotypes, genes and mutation rates associated with MCDs, using the key words “microcephaly”, “megalencephaly”, “lissencephaly”, “polymicrogyria”, “schizencephaly”, “cobblestone malformation”, “focal cortical dysplasia” and “heterotopia”. The most recent search was performed on 31 October 2019.

From the MCD expert laboratories within the Neuro-MIG network, headed by M.W., K.S., U.H., E.P. and N.D.D., we collected data regarding gene panels, enrichment strategies and diagnostic yield. Using the data obtained as described above, we compiled lists of genes associated with the various MCD subtypes and defined a diagnostic strategy for patients with MCDs. The gene list was curated — that is, checked, corrected and completed — by all authors on the basis of long-standing personal experience gained through molecular diagnostics in patients with MCDs. The first draft was finalized before the second meeting. During the first round of voting, 21 of the authors voted on 101 recommendation statements. Agreement (>90% positive votes) was reached for 89 statements, and the remaining 12 were revised according to the reasons provided for disagreement. The second round of voting involved 42 experts. At the end of the process, 94 recommendations found >90% consensus. In addition, five statements were agreed on by 80–90%, two statements by 75–80% and one statement by 70–75% of the participants (Supplementary Table 1). Recommendations with consensus <80% were excluded from the recommendations section below. Unless specified otherwise, we report on recommendation statements with >90% consensus.

## Clinical presentation of MCDs

MCDs can be isolated or associated with a wide variety of neurological and extra-neurological features, including other birth defects and facial dysmorphism. The age at clinical referral and the severity of neurological deficits vary substantially between affected individuals. The most common presenting features are epilepsy, developmental delay and/or motor abnormalities of tone, movement and posture^1^. These features are listed in relation to the typical ages of presentation in Box 1.

Box 1 Common presentation of MCD **Fetal**

- Reduced fetal movements
- Polyhydramnios
- Ultrasound and/or MRI abnormalities

**At birth**

- Microcephaly or macrocephaly
- Dysmorphic features
- Congenital abnormalities
- Abnormal muscle tone
- Feeding difficulties
- Breathing difficulties
- Cranial ultrasound, MRI and/or CT abnormalities

**Infancy**

- Global developmental delay
- Hypotonia or hypertonia
- Feeding difficulties
- Postnatal microcephaly or macrocephaly
- Cerebral palsy
- Epilepsy including infantile spasms
- MRI and/or CT abnormalities

**Childhood**

- Cerebral palsy
- Seizures
- Speech delay
- Cognitive delay
- Drooling and/or congenital suprabulbar paresis
- Visual defects
- Ocular motor apraxia
- MRI and/or CT abnormalities

**Adolescence, adulthood**

- Epilepsy
- Intellectual disability
- Hypotonia or hypertonia
- MRI and/or CT abnormalities

## Main MCD types

**Fig. 1:**
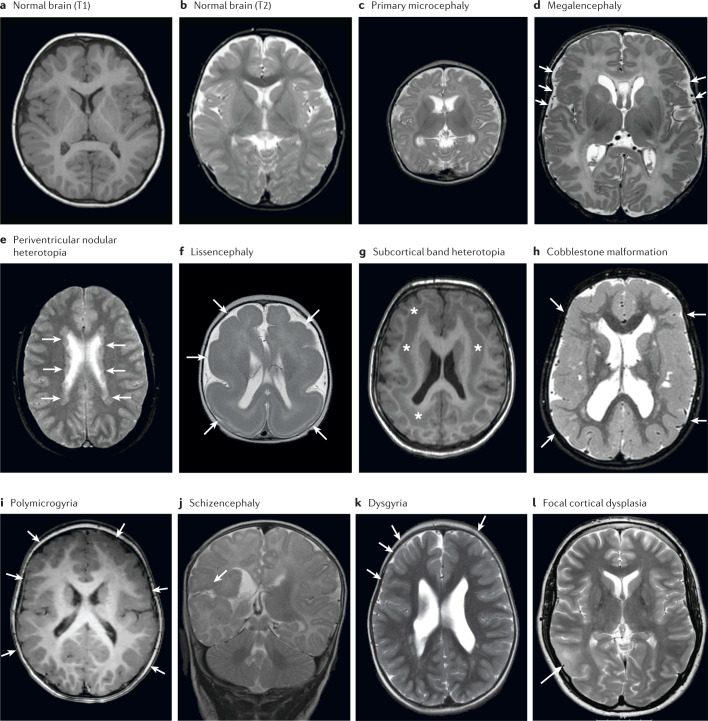
MRI scans showing common malformations of cortical development. The brain was scanned in the axial plane unless otherwise stated. **a** | Normal brain on T1-weighted images. **b** | Normal brain on T2-weighted images. **c** | Primary microcephaly with a small brain. **d** | Abnormally large brain (megalencephaly) with abnormal appearance of the perisylvian cortex (arrows point to small gyri suggestive of polymicrogyria). **e** | Bilateral nodular heterotopia (arrows) situated along the ventricular walls. **f** | Lissencephaly spectrum with agyria–severe pachygyria (arrows). **g** | Lissencephaly spectrum with subcortical band heterotopia visible as a thick band isointense to the cortex (asterisks). **h** | Generalized thickened cortex with broad gyri and white matter abnormalities consistent with cobblestone complex (arrows). **i** | Bilateral frontoparietal polymicrogyria with abnormally small gyri and shallow sulci (arrows). **j** | Coronal scan showing schizencephaly, characterized by a cleft lined by grey matter extending from the cortex to the ventricle (arrow). **k** | Abnormally oriented sulci of varying depth with normal cortical thickness (arrows). **l** | Focal cortical dysplasia with blurring of the grey–white matter boundary and hyperintensity of the white matter on T2-weighted imaging (arrow).

In this section, we provide an overview of the most common types of MCD and their aetiologies. Different descriptions have been introduced in the literature over the years depending on the study design and the medical background of the research group (for example, neurologists, radiologists, geneticists or pathologists). Table 1 summarizes the consensus definitions that were agreed on by our working group. These definitions are used throughout the text, and brain imaging examples are provided in Fig. 1. The descriptions are specific to each term and do not consider the presence of abnormalities of other brain structures, which often coexist with MCD. Each MCD type can be further classified on the basis of morphology, topography, severity gradient and involvement of other brain structures^1^. A detailed paper on the MCD neuroimaging features has been published separately by representatives from the Neuro-MIG network^8^.

**Table 1:** Consensus definitions of the main MCD types

Phenotype	HPO ID	Description
Microcephaly	HP:0000252	A significant reduction in OFC by ≥2 s.d. ^a^compared with controls matched for age and sex^9,10^
Megalencephaly	HP:0001355	A significant increase in OFC, and specifically brain size, by ≥3 s.d. compared with controls matched for age and sex^b^
Periventricular nodular heterotopia (PVNH)	HP:0032388	Grey matter nodules along the ventricular walls^1^
Lissencephaly spectrum	HP:0001339	Includes agyria, pachygyria and subcortical band heterotopia
Agyria, pachygyria	HP:0031882, HP:0001302	Abnormal gyral pattern with absent or broad gyri in combination with an abnormally thick cortex^18^
Subcortical band heterotopia (SBH)	HP:0032409	A band of grey matter separated from the cortex and lateral ventricles by zones of white matter^18^
Cobblestone malformation (COB)	HP:0007260	An irregular and ‘pebbled’ cerebral surface with moderately thick cortex and jagged grey–white matter border with frequent vertical (perpendicular to the cortex–white matter border) striations^22,23^
Polymicrogyria	HP:0002126	An excessive number of abnormally small cerebral gyri with cortical overfolding, irregular ‘pebbled’ cortical surface and a ‘stippled’ grey–white matter boundary^28^
Schizencephaly	HP:0010636	A full-thickness cerebral cleft lined with grey matter, which extends from the ventricular surface to the pial surface^174^
Focal cortical dysplasia (FCD)	HP:0032046	Cortical dyslamination, with or without abnormal cell types (dysmorphic neurons and balloon cells). Other features can include gyral and/or sulcal irregularities; increased cortical thickness; blurring of the cortex–white matter junction; and white matter abnormalities, such as increased signal on T2-weighted images or a radially oriented ‘transmantle sign’ of T2 hyperintensity extending from the abnormal cortex to the lateral ventricle^171^
Dysgyria	HP:0032398	A cortex of variable thickness and a smooth grey–white boundary but with an abnormal gyral pattern characterized by irregularities of sulcal depth and or orientation^30,31^. This term is only used to characterize cortical malformations that do not meet the classic features of any of the abovementioned subtypes

Examples of imaging findings in these conditions are provided in Fig. 1. HPO ID, Human Phenotype Ontology identifier; MCD, malformation of cortical development. ^a^Some studies define microcephaly as occipitofrontal circumference (OFC) ≥3 s.d. below the mean, referring to OFC 2–3 s.d. below the mean as borderline microcephaly. ^b^Megalencephaly specifically refers to a brain size that is ≥3 s.d. above the mean and is primarily a developmental brain disorder, whereas macrocephaly (defined as an OFC ≥3 s.d. above the mean) has a wide variety of causes besides megalencephaly, including ventriculomegaly, hydrocephalus and increased skull thickness.

### Microcephaly

Microcephaly is defined as a significant reduction in the occipitofrontal circumference (OFC) compared with controls matched for age and sex. Microcephaly is the most common MCD and is present in 15% of children referred for evaluation of developmental disabilities^9^. The relevant degree of reduction differs throughout the literature, being set at 2–3 s.d. below the mean^9–12^. Strictly speaking, microcephaly is a clinical finding rather than a disease; however, it provides a reliable estimation of the brain volume^10^. The final brain size is the result of a complex process of neural stem cell proliferation, migration, and ongoing organization, synaptogenesis and apoptosis^11^. Microcephaly is classed as congenital if present at birth (primary microcephaly) or postnatal if it develops after birth (secondary microcephaly)^10,13,14^. These two groups also have different molecular aetiologies^11^. Microcephaly can present with a normal or simplified gyral pattern, or with additional, more complex brain abnormalities^11^. The clinical outcome cannot be predicted by head size alone and largely depends on the underlying cause and the appearance of the brain on MRI.

### Macrocephaly and megalencephaly

Macrocephaly is defined as an OFC ≥2 s.d. above the mean, whereas megalencephaly refers to an abnormally large brain size^1^. Macrocephaly has a wide variety of causes besides megalencephaly, including hydrocephalus and increased skull thickness. Mild megalencephaly (2–3 s.d. above the mean) with an otherwise structurally normal brain can be seen in typically developing children, often in the setting of benign familial macrocephaly^15^. However, megalencephaly can point to an underlying neurodevelopmental or generalized overgrowth disorder.

### Periventricular nodular heterotopia

The term neuronal heterotopia refers to groups of neurons in an abnormal location, and periventricular nodular heterotopia (PVNH) describes nodular masses of grey matter located along the ventricular walls protruding into the ventricle^1^. PVNH can occur in isolation or together with other brain or body malformations and is not rare: in one study, PVNH was observed in 0.48% of the general paediatric population^16^. The nodules can occur unilaterally or bilaterally, and should be further defined according to their number and location (for example, involving the frontal or temporal and/or occipital horns of the lateral ventricles).

PVNH is associated with numerous different copy number variations (CNVs) and single gene variants, and can be part of a complex syndromic disorder.

### Lissencephaly spectrum

The lissencephaly spectrum encompasses agyria, pachygyria and subcortical band heterotopia (SBH)^17^. Agyria and pachygyria are characterized by an abnormal gyral pattern with absent gyri (agyria) or broad gyri (pachygyria) in combination with an abnormally thick cortex^18^. SBH describes a band of grey matter separated from the cortex and lateral ventricles by zones of white matter^18^. In rare cases, pachygyria and SBH can co-occur in the same brain, with a typical pattern of frontal pachygyria and posterior SBH^19^. Microlissencephaly represents a separate subgroup and is defined as a combination of lissencephaly (usually in the form of agyria or pachygyria) with severe congenital microcephaly (OFC at birth ≥3 s.d. below the mean)^20^.

### Subcortical heterotopia

Subcortical heterotopia (SUBH) refers to brain malformations with clusters of neurons located within the white matter, between the cortex and lateral ventricles^21^. The well-recognized and aforementioned PVNH and SBH have distinct imaging patterns and are classified separately. Multiple terms have been used to describe this type of malformation, including giant, curvilinear, nodular, focal and massive heterotopias^21^. In 2019, a group within the Neuro-MIG network provided the first framework for an imaging classification of SUBH that encompasses five groups further subdivided into specific entities^21^.

### Cobblestone malformation

COB is recognized as an undersulcated, irregular and ‘pebbled’ cerebral surface, with a moderately thick cortex^22,23^. This malformation is caused by defects of the pial limiting membrane with resulting neuronal overmigration from the cortical plate into the leptomeninges^3,24^. COB often co-occurs with eye, muscle and additional brain malformations within the spectrum of the α-dystroglycanopathies, with Walker–Warburg syndrome at the most severe end^25^.

COB was originally described as lissencephaly type 2 but this term has now been abandoned^26^. In addition, COB is often confused with polymicrogyria^27^. The strict differentiation of COB-related and polymicrogyria-related genes in the literature remains difficult, as several conditions characterized by COB were reported as polymicrogyria-associated disorders (for example, *GPR56*-associated frontoparietal ‘polymicrogyria’ and CHIME syndrome).

### Polymicrogyria

Polymicrogyria is one of the most frequent types of MCD and is also one of the most heterogeneous in aetiology^1^. Polymicrogyria is defined as an excessive number of abnormally small cerebral gyri with cortical overfolding, an irregular, pebbled cortical surface and a stippled grey–white matter boundary^28^.

As highlighted in the previous section, polymicrogyria can be difficult to differentiate from COB, and might also be confused with dysgyria or pachygyria. High-resolution imaging can aid the differentiation of these conditions, as it can show microgyri, microsulci and stippling of the grey–white matter junction — a specific feature of polymicrogyria that is not seen in other MCDs^1^. Of note, the Sylvian fissures, which are best viewed on sagittal imaging, should be closely scrutinized as polymicrogyria often affects these areas preferentially, with abnormal posterior extension and sulcal branching being observed^28^. Polymicrogyria is frequently seen in association with many other brain malformations and is sporadically described in various syndromic disorders. Polymicrogyria has been classified into six topographic patterns that are further divided into 13 morphological subtypes^28^. Moreover, at least six polymicrogyria syndromes have been defined on the basis of radiological and clinical features^29^.

### Dysgyria

Dysgyria translates as abnormal gyration and can therefore be applied to almost every type of MCD. However, this term was introduced to describe cortical malformations that do not meet classic features of any of the abovementioned well-established MCD types. Dysgyria describes a cortex of variable thickness and an abnormal gyral pattern characterized by abnormalities of sulcal depth or orientation (for example, obliquely oriented sulci directed radially towards the centre of the cerebrum and narrow gyri separated by abnormally deep or shallow sulci)^30,31^. In the vast majority of cases, the term dysgyria describes an abnormal non-lissencephaly, non-polymicrogyria cortex within the spectrum of tubulinopathies.

### FCD and hemimegalencephaly

FCD is identified on brain imaging by focal irregularities of cortical morphology and thickness, blurring of the grey–white matter boundary, and white matter T2 hyperintensity. Depending on the size of the lesion and the resolution of the brain imaging, FCD can be missed on MRI. Smaller lesions are often only identified on neuropathological studies after surgery for epilepsy. FCD type II is characterized by the presence of dysplastic, megalocytic neurons, a feature that is also present in hemimegalencephaly. Balloon cells are also observed in FCD IIB and hemimegalencephaly^32^. The size of the lesion varies from submicroscopic involvement of one or several sulci (FCD) to a larger area involving a lobe (partial hemimegalencephaly) or involvement of an entire cerebral hemisphere (classic hemimegalencephaly)^32^. In the latter condition, the affected hemisphere is visibly enlarged. In hemimegalencephaly, the lesion can extend to non-brain tissue, and clinicians should look out for skin abnormalities and localized overgrowth of one or several body parts.

## Molecular testing: current practice

### Chromosomal testing

MCDs have been linked to a wide range of CNVs, as detected by chromosomal microarray analysis (CMA)^1,33,34^. Several CNVs are consistently associated with MCD, the most common of which are the 22q11 and 1p36 deletions associated with polymicrogyria, the 17p13.3 deletion (encompassing *LIS1* (also known as *PAFAH1B1*), *YWHAE* and other genes) that causes Miller–Dieker syndrome and isolated lissencephaly, and 6qter deletions associated with various brain malformations including polymicrogyria and PVNH^33,35,36^. A study published in 2019 reported a diagnostic yield of 36% when CMA was used in patients who had PVNH with or without other malformations, and 9% in a group with polymicrogyria only^37^. Another study did not show an increased burden of rare CNVs in people with polymicrogyria compared with healthy controls^38^. In patients with microcephaly, the yield was ~5–7%^13,39^. In a large cohort of patients with lissencephaly (*n* = 811), Miller–Dieker syndrome was diagnosed in 9% of cases^40^. Several MCD-related genes frequently harbour intragenic deletions or duplications, which might be identified by standard microarrays^41–43^.

### Single gene testing

Single gene testing is being superseded by NGS gene panels, and we were only able to identify systematic studies for a small number of MCD types. The yield of single gene testing varies greatly depending on the MCD type and extension of the malformation. For SBH, the yield of molecular testing is high, with pathogenic variants in *DCX* or *LIS1* being found in 79% of patients (123 of 155)^40^. Pathogenic variants in *FLNA* are important aetiological factors for PVNH. The highest frequency is found in women with bilateral frontocentral PVNH, especially in combination with cerebellar hypoplasia and/or mega cisterna magna, with a positive family history of PVNH^44,45^. The yield varies from 80–100% in female familial cases to 9–26% in sporadic cases^44–46^.

In a cohort of 113 patients with MCDs, a molecular diagnosis was established in 21 patients (19%) by targeted testing of one or more genes selected on the basis of the phenotype^4^. In a more recent study consisting of an Argentinian cohort of 38 patients with lissencephaly, SBH or PVNH, pathogenic variants were identified in 36% of cases^46^.

Pathogenic variants of *ASPM* are the most common genetic cause of primary microcephaly, with a mutation rate of 10–40% depending on ethnicity and the presence or absence of consanguinity^47,48^. Among consanguineous families, alterations in *ASPM* and *WDR62* accounted for >50% of cases of primary microcephaly^49,50^.

For COB, mutation detection rates vary considerably, depending on the age at diagnosis and clinical inclusion criteria. For the most severe prenatal manifestations, the detection rate was usually >60% when the six genes most commonly linked to dystroglycanopathy were analysed^25^.

### Gene panels

Despite multiple publications reporting on the yield of gene panels in cohorts of patients with neurodevelopmental disorders^51–53^, similar studies for MCDs are scarce. The only study that we identified reported on testing of a small gene panel (ten genes) in 158 individuals with brain malformations, including 30 individuals with SBH, 20 with megalencephaly, 61 with PVNH and 47 with pachygyria. Causal pathogenic variants were found in 27 individuals (17%, range 10–30% depending on the phenotype)^54^.

Several genes encoding components of the PI3K–AKT–mTOR pathway have been implicated in FCD, and targeted testing of PI3K–AKT–mTOR pathway genes, using highly sensitive sequencing methods that allowed detection of low-frequency brain somatic variants, produced diagnostic yields ranging from 12% to 40%^55–57^. In a different cohort, a targeted NGS panel that included the most commonly mutated PI3K–AKT–mTOR pathway genes uncovered *PIK3CA* pathogenic variants in 50 of 131 individuals (174 samples) with the megalencephaly–capillary malformation syndrome^58^.

### Exome sequencing

One study investigated the yield of exome sequencing, combined with CMA, in 54 patients with various MCD types^5^. This approach yielded a definitive (9/16) or presumptive (7/16) molecular diagnosis in 16 of 54 enrolled individuals (30%). Another study of 62 patients with microcephaly followed a similar approach and identified causative variants in 48% of the individuals^39^.

### Neuro-MIG laboratories

We have also analysed the yield from the diagnostic laboratories within the Neuro-MIG network. Targeted gene panels resulted in diagnostic yields of 15–37%, although wide variability was observed among the different clinical subtypes (Table 2). The combination of expert evaluation of MRI scans followed by targeted analysis of the most plausible causative variants can considerably increase the diagnostic yield. Substantiating this point, the availability of MRI scans resulted in an improved mutation detection rate of 37% in a mixed cohort of 117 patients with MCDs, compared with only 18% in a cohort of 784 patients analysed without previous expert re-evaluation of MRI scans at the Human Genetics Center Regensburg (U.H., unpublished work). In the former cohort, the testing strategy was selected by the laboratory depending on the MRI pattern, and the approaches included single gene, panel and exome sequencing. A similar trend was noted in the Department of Clinical Genetics, Erasmus MC University Medical Center, Rotterdam, where the diagnostic yields from in-house requests accompanied by expert MRI review by G.M.S.M. were almost double those from the tests ordered from other medical specialists outside the university hospital (M.W., unpublished work).

**Table 2:** Diagnostic yield across Neuro-MIG

MCD entity	Diagnostic yield (%)^a^
Microcephaly^b^	18–20
Lissencephaly	75–81
Cobblestone malformation	75
Polymicrogyria	20
Periventricular nodular heterotopia	30–37
Total cohort (*n* = 737)	15–37

The data were collected during the Neuro-MIG network expert meeting in St Julians, Malta (21–23 February 2018) and represent the unpublished internal diagnostic yield after the introduction of next-generation sequencing in clinical routine. The diagnostic yield per malformation was not provided by every laboratory; data on cobblestone malformation and periventricular nodular heterotopia were only available from the Center for Human Genetics Regensburg, Germany (U.H., unpublished work). MCD, malformation of cortical development. ^a^Quoted figures are for class 4 (likely pathogenic) and class 5 (definitely pathogenic) variants. ^b^Note that diagnostic yield is increased in patients with microcephaly defined as 3 s.d. below the mean.

### In utero infections

Prenatal infections can cause extensive damage to the fetal brain, including the cerebral cortex^59–61^. Cytomegalovirus (CMV) is one of the most frequent non-genetic causes of MCDs and is specifically associated with polymicrogyria, intracranial calcifications, white matter abnormalities and microcephaly^1^. In a cohort of 26 patients with bilateral polymicrogyria, six (31%) tested positive for CMV; however, it was unclear whether these patients were infected prenatally or postnatally^62^. In a larger group of 50 patients with polymicrogyria, six (12%) tested positive on Guthrie cards (W.B.D., unpublished work).

In one study of 41 newborn babies with symptomatic CMV, eight (19.5%) presented with microcephaly^63^. Not all CMV-infected individuals are symptomatic at birth, and neurological sequelae can develop later in life^64^. Other infectious agents, including rubella virus^65^, varicella zoster^66^ and herpes simplex virus^61,67^, can also cause microcephaly. In recent years, Zika virus has been associated with primary microcephaly and a spectrum of brain malformations^68–74^.

## New recommendations

The Neuro-MIG network recommends that a concerted effort be made to reach an aetiological diagnosis in every individual with an MCD. The diagnosis serves several functions. First, it explains the cause of the malformation, ends the diagnostic odyssey and prevents further unnecessary investigations. Second, it provides information on prognosis and recurrence risk for the patient and family members^4^. Third, it aids the prediction of treatment outcomes; for example, the success rate for epilepsy surgery depends on the underlying genetic cause^75^. Fourth, it directs patient management (for example, antiviral treatment and screening for progressive hearing loss in infants with congenital CMV infection^76^, cardiovascular surveillance in *FLNA*-related and *ARFGEF2*-related PNVH^77,78^ or mTORC1 inhibition in patients with tuberous sclerosis complex (TSC))^79^. Fifth, it enables natural history studies^80,81^ and targeted research into personalized therapy and prevention^82,83^.

**Fig. 2:**
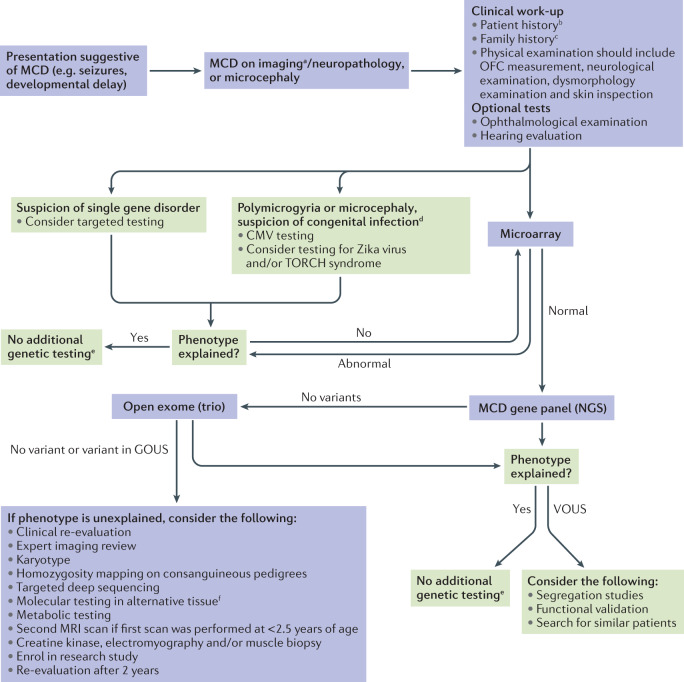
Diagnostic workflow for MCDs. This step-by-step diagnostic approach was formulated by Neuro-MIG. The main diagnostic steps are in purple-lined boxes. ^a^Seek expert review. ^b^Including prenatal and perinatal history. ^c^Includes construction of a pedigree and enquiry for consanguinity. ^d^Based on additional features (for example, sick infant, abnormal liver function tests, retinal scarring or hearing loss), perinatal history (for example, maternal rash or fever) and/or imaging abnormalities (for example, calcifications, white matter injury or cysts). ^e^Offer genetic counselling and segregation analysis to the patient and family members. ^f^Affected brain tissue (if available), fibroblasts or saliva. CMV, cytomegalovirus; GOUS, gene of uncertain significance; MCD, malformation of cortical development; NGS, next-generation sequencing; OFC, occipitofrontal circumference, VOUS, variant of uncertain significance.

Imaging findings, such as generalized versus focal and bilateral versus unilateral malformations, cannot reliably distinguish genetic from non-genetic causes, and the diagnostic yield of targeted testing is determined to a large extent by the availability of a multidisciplinary expert evaluation. However, such an ideal setting can rarely be met in practice. Therefore, we have formulated a general diagnostic workflow that can be applied in most clinics to any individual with an MCD (Fig. 2). Lists of currently known MCD-associated genes are presented in Supplementary Tables 2 and 3. These lists can assist variant interpretation and guide targeted testing if exome (or genome) sequencing is not available. These general recommendations should minimize the chance of missing a known causative variant. The workflow can be started when a person is first diagnosed with an MCD, although clinicians should check whether any of the investigations have already been performed.

For some MCD subtypes, the most cost-effective strategy would be targeted gene analysis, but the success of this approach depends greatly on accurate pattern recognition. The relevant subtype-specific patterns and aetiologies are outlined in the section ‘Phenotype-specific considerations’ below.

The correct interpretation of genetic test results requires detailed phenotypic analysis, including re-evaluation of the brain MRI, to confirm that the identified single nucleotide variant (SNV) or CNV fully explains the phenotype. In the case of a negative result, the re-evaluation should help determine whether the malformation was correctly classified, whether additional diagnostic testing, such as deep sequencing or analysis of a different tissue, might be helpful, and whether a non-genetic cause is more likely.

We recommend that a final clinical interpretation is done by a qualified medical geneticist, preferably after an interdisciplinary discussion with a molecular geneticist, neuroradiologist and/or neurologist. Unusual cases can be presented at an expert review session. Selected case reports demonstrating the importance of phenotype-guided interpretation of the test results are summarized in Supplementary Box 1.

### Strategy if no diagnosis is reached

If no diagnosis has been reached after the general workflow has been applied, several strategies can be considered.

Patients with an MCD pattern that is known to be highly specific for one or a few genes could benefit from visual inspection of NGS reads and/or alternative targeted sequencing methods such as Sanger sequencing complemented by deletion/duplication testing of genes of interest^84^, as outlined in the section ‘Phenotype-specific considerations’ below. Review of NGS data might reveal inadequate coverage of the genes of interest, or the filtering out of potentially relevant splice site or flanking intronic sequences.

If not performed previously, karyotype analysis should be considered in undiagnosed patients with MCDs (86% consensus from the Neuro-MIG network). Balanced translocations and ring chromosome abnormalities are a rare cause of MCDs but have occasionally been described^35,85^.

Patients from consanguineous pedigrees and families with multiple affected siblings might benefit from a single nucleotide polymorphism microarray analysis to identify regions of homozygosity. If a homozygous region contains a known MCD-related gene that is compatible with the phenotype, special attention must be given to the known deep intronic variants^86–89^ (listed in Supplementary Table 3).

Metabolic investigations should be considered in patients with microcephaly, polymicrogyria or COB, as a broad range of metabolic diseases, including peroxisomal disorders, glutaric aciduria, fumarase deficiency and D-bifunctional protein deficiency, can manifest with cortical malformations resembling these MCD patterns^1^.

In patients with unexplained MCDs and muscle weakness and/or elevated creatine kinase, a muscle biopsy might be considered to allow specific analysis for dystroglycanopathies and mitochondrial disorders. The results of muscle biopsy allied to characteristic brain imaging findings in the CNS may help to indicate the affected gene^90^.

Some patients might benefit from repeat brain imaging, especially if the first MRI scan was performed before completion of myelination (3 months to 2.5 years of age) or was of low quality (for example, low resolution, or inadequate exploration of the brain according to the axial, coronal and sagittal plan and/or inadequate sequences). Occasionally, brain MRI scans of the parents can identify a previously unrecognized familial malformation syndrome^41,91,92^.

Autopsy represents an important final procedure in deceased patients with unexplained MCDs as it can provide additional information that cannot be obtained during life^93^. Also, after brain surgery, DNA can be extracted from affected brain tissue to identify somatic pathogenic variants. Specific protocols are recommended for the evaluation of perinatal and postnatal brain tissue, including both frozen and fixed tissue samples from key brain regions (that is, regions that are vulnerable to epilepsy-related damage) to identify specific structural abnormalities and rule out other pathologies^94^.

Finally, patients without a diagnosis should be considered for trio-based whole-genome sequencing and RNA sequencing, preferably within a large collaborative research network to allow rapid discovery of novel causative variants, non-coding variants in regulatory elements and epigenetic variations^95–97^.

### Recurrence risk and genetic counselling

Only when the cause of the MCD is known can an accurate recurrence risk be provided to the patient and their family. When the cause is unknown, an attempt should be made to provide an empirical risk figure. This figure depends on the type of malformation, clinical presentation and the causes that have been reliably excluded (Table 3). We should point out that empirical risk counselling requires very high confidence in correct MRI interpretation and recognition of the specific phenotype.

**Table 3:** MCD empirical recurrence risk

MCD entity	Known inheritance patterns^a^	General empirical recurrence risk
Microcephaly	AD, AR, XL, non-Mendelian (imprinting, mitochondrial), non-genetic	No reliable estimate available; all inheritance patterns should be discussed
Megalencephaly	AD, AR, XL, non-Mendelian (imprinting, mitochondrial, postzygotic mosaic)	No reliable estimate available; all inheritance patterns should be discussed Low for siblings if clinical presentation in proband is highly suggestive of a mosaic disorder
Lissencephaly: cortex >10 mm	AD, rarely XL or AR	Probably low for siblings Caution especially in families with consanguinity; recessive inheritance has been reported^175,176^
Lissencephaly: cortex 5–10 mm	AR, AD (tubulinopathy)	Risk for siblings 25% unless phenotype is classified as tubulinopathy (AD) Risk for offspring depends on the carrier status and/or degree of family relationship with the partner (up to 50% if partner is a carrier)
Lissencephaly: subcortical band heterotopia (SBH)	XL (diffuse SBH) or mosaic	XL risk for siblings — discuss up to 50% as mother can be an asymptomatic carrier Risk for offspring 50% (≤50% if postzygotic mosaic is suspected); males are not known to reproduce
Cobblestone malformation (COB)	AR	Risk for siblings 25%
Periventricular nodular heterotopia (PVNH)	XLD, AD, AR, non-genetic	No reliable estimate available; all inheritance patterns should be discussed; probably low risk for single nodules
Subcortical heterotopia (SUBH)	Minority AR, most unknown, possible non-genetic or postzygotic mosaic	Risk for siblings probably low unless AR disorder is clinically recognized (25%) Risks for offspring probably low (no vertical transmission documented to date)
Polymicrogyria	AD, AR, XL, non-genetic	No reliable estimate available; consider that polymicrogyria is easily confused with COB
Tubulinopathies	AD	If parents are unaffected, risk for siblings is low Risk for offspring ≤50%
Focal cortical dysplasia (FCD) and hemimegalencephaly	Postzygotic mosaic, AD with or without reduced penetrance	Probably low for single isolated cases or if no germline variants in *TSC1*, *TSC2*, *DEPDC5*, *NPRL2* or *NPRL2* have been identified; otherwise up to 50%

AD autosomal dominant; AR autosomal recessive; MCD, malformations of cortical development; XL, X-linked; XLD, X-linked dominant. ^a^Additional inheritance patterns might be discovered in the future.

### Phenotype-specific considerations

#### Microcephaly

The aetiology of microcephaly is heterogeneous and includes both genetic and non-genetic factors. Non-genetic causes, including intrauterine teratogen exposure (for example, alcohol or drugs), congenital infections and perinatal and postnatal brain injuries (placental insufficiency, birth complications, postnatal infarcts and concussions), account for almost 30% of microcephaly cases. Recognized genetic causes include chromosomal aneuploidies, CNVs, some of which are submicroscopic, and a rapidly growing number of single gene disorders (reviewed by Pirozzi et al.^11^). Accurate perinatal history-taking aids the identification of teratogen exposure and infections, although a negative history can never reliably rule out these causes. Brain scans should be scrutinized for signs of fetal injury, including gliosis, cysts and calcifications. Clinicians should be aware that cortical malformations, especially polymicrogyria, can also be caused by fetal injury (see also below). Recurrence in the family, dysmorphic features and congenital abnormalities outside the CNS can be indicative of a genetic cause.

Ophthalmological abnormalities are found in up to 48% of patients with microcephaly^98,99^, including chorioretinal lacunae in Aicardi syndrome, chorioretinopathy in *KIF11*-related microcephaly, microphthalmia and cataract in Warburg Micro syndrome and cerebro-oculo-facio-skeletal syndrome, chorioretinitis after in utero CMV or toxoplasmosis infection, and a wide spectrum of abnormalities of the macula, retina and optic nerve after in utero Zika virus infection. Therefore, a detailed eye examination should be routinely performed in every individual with microcephaly so that appropriate support and diagnostics can be implemented.

#### Megalencephaly

Examination of an individual with megalencephaly should include an assessment of whether the malformation is confined to the brain or whether it is associated with a generalized or segmental overgrowth syndrome. Careful assessment of serial height, weight and OFC measurements is helpful, as is examining the body for any asymmetries and skin abnormalities. Overgrowth usually manifests within the first 2 years of life^100^. Currently, >20 generalized overgrowth syndromes are known (reviewed elsewhere^100,101^). Distinctive facial features can also aid identification of the underlying syndrome.

Generalized overgrowth syndromes are most often caused by germline gene mutations or CNVs, which can be identified with the standardized workflow. By contrast, segmental overgrowth syndromes and some isolated megalencephaly syndromes are caused by somatic mutations that might elude detection by standard workflows. To increase the chance of identifying the disease-causing variant, it might be necessary to sequence DNA derived from affected tissue (for example, skin or brain specimens) instead of blood. Further details of this approach are provided in the section ‘Detecting mosaic variants’ below. Several overgrowth syndromes, as well as the PTEN hamartoma tumour syndrome, are associated with an increased risk of malignancies.

An increasing number of defects in genes involved in cell growth and proliferation pathways are being identified in megalencephaly. The affected pathways and molecules include the PI3K–AKT–mTOR and RAS–MAPK–ERK pathways, DNA methyltransferases, transcription initiation regulators and receptor tyrosine kinases^11,102,103^. In our experience, PI3K–AKT–mTOR pathway-associated megalencephaly is often ≥3 s.d. above the mean. Mutations in this pathway can cause either isolated or syndromal megalencephaly, with other features including somatic (body) overgrowth and/or other MCDs, including polymicrogyria^104,105^. Given the high prevalence of mosaicism in these disorders, a tailored approach is recommended (see below).

#### Lissencephaly spectrum

The lissencephaly imaging classification was updated in 2017 and now includes 21 patterns^17^. Lissencephaly is considered to be an exclusively genetic disorder^40^, with 28 genes currently known to be associated with this condition (Supplementary Table 3). Four lissencephaly patterns are highly specific for pathogenic variants in one or two genes, with diagnostic yields >90%^40^. The first pattern is diffuse agyria with cortical thickness >10 mm, which is caused by *LIS1* and *DCX* variants. The main cause in this group is a microdeletion at chromosome 17p13.3, the *LIS1* locus, which can cause isolated lissencephaly, or Miller–Dieker syndrome in the case of a larger deletion^40^. The second specific pattern is occipital agyria combined with frontal pachygyria, which is primarily associated with deletions and pathogenic variants in *LIS1*, but also in rare cases with *TUBG1* variants and *TUBA1A* variants affecting codon Arg402. The third pattern is pachygyria with a cortical thickness of 5–10 mm, most prominent over the temporal lobes, combined with complete agenesis of the corpus callosum and severe hypomyelination. This pattern is caused by *ARX* pathogenic variants. Note that pathogenic variants in *DYNC1H1* have been linked to a similar lissencephaly pattern but without hypomyelination. The fourth pattern, diffuse SBH with a band thickness >5 mm, is a pathognomonic pattern strongly associated with pathogenic variants in *DCX* in both women and men^40^. Posterior-predominant SBH is associated with mild or mosaic *LIS1* mutations^40^.

No other genes have been associated with these patterns. Therefore, a negative test result for those genes in a patient with a specific phenotype should prompt an offer to the family to participate in a research project focusing on gene discovery.

#### Periventricular nodular heterotopia

PVNH is associated with numerous CNVs and single gene mutations and can be part of a complex syndromic disorder, such as van Maldergem syndrome, Donnai–Barrow syndrome, Au–Kline syndrome or Noonan-like syndrome with loose anagen hair^37^. Proteins encoded by the genes associated with PVNH are involved in several cellular and molecular mechanisms, including the formation of the radial glial scaffold, cell–cell adhesion and vesicle trafficking. In addition, dysregulation of PI3K–AKT–mTOR or SMAD2/3 signalling pathways, RNA processing or transcriptional regulation has been reported in people with PVNH^106–108^. At least 20 genes have been associated with this condition (Supplementary Table 2).

*FLNA* mutations are an important monogenic cause of PVNH and, owing to a substantial risk of cardiovascular and other organ complications, identification of *FLNA*-related disorders is of great clinical importance^77,109^. Although no single feature is pathognomonic, several features should raise suspicion of an *FLNA* mutation, including female sex, with or without a positive family history that follows an X-linked dominant pattern; absence of overt intellectual disability, although learning difficulties, dyslexia and/or psychiatric problems can be present^110,111^; bilateral clusters of confluent nodules extending along the walls of the frontocentral lateral ventricles (classic PVNH)^44^; and the presence of a retrocerebellar cyst or mega cisterna magna^44,110^. Less frequently, corpus callosum hypoplasia, inward rotated anterior ventricular horns, white matter abnormalities and/or focal cortical abnormalities can be observed^77,110^. Systemic involvement is not an obligatory feature but can be present, leading to cardiovascular abnormalities such as patent ductus arteriosus, aortic aneurysm and cardiac valvular dystrophy; obstructive lung disease; constipation; coagulopathy; joint hypermobility; and other connective tissue abnormalities^77,109,110^.

In individuals with one or two single nodules, normal cognitive functioning and no other congenital abnormalities, the yield of genetic testing is low. However, these individuals can harbour mosaic *FLNA* mutations that might be passed on through the germline to their offspring^44^.

Posterior-predominant PVNH is a common pattern that is often associated with overlying polymicrogyria and/or subcortical heterotopia, as well as abnormalities of the fossa posterior, corpus callosum and/or hippocampus^112^. This pattern can be caused by a microdeletion of chromosome 6q27, but has also been associated with fetal brain injury^36,113^.

#### Subcortical heterotopia

Several rare, mostly symmetrical bilateral forms of SUBH have a genetic origin, usually with an autosomal recessive mode of inheritance. Extensive brain involvement is seen in the mesial parasagittal form associated with Chudley–McCullough syndrome, which results from biallelic variants in *GPSM2*, and ribbon-like heterotopia, in combination with agenesis of the corpus callosum and megalencephaly, is observed in individuals with biallelic *EML1* variants^114,115^. Another rare subtype affecting the peritrigonal regions has been observed in patients with variants in genes encoding a microtubule component (*TUBB*), a microtubule-severing protein that localizes to the centrosome and mitotic spindle during cell division (*KATNB1*), or a centrosomal protein with tubulin-dimer binding activity (*CENPJ*)^21^.

In parallel with the diverse morphology of SUBH, the aetiology of this condition is also very heterogeneous, and for certain subtypes is largely unknown. For example, no genetic cause has been identified for curvilinear heterotopia, which is often asymmetric and can extend from the cortex to the ependyma^21,116^. However, a vascular disruptive cause has been suggested in several patients on the basis of a prenatal history of twinning, near miscarriage or trauma^117–120^, and some cases are hypothesized to result from postzygotic mutations^21^.

#### Polymicrogyria

The aetiology of polymicrogyria can be either genetic or disruptive^27^, and our new clinical workflow has been designed to make the physician aware of potential pitfalls. Despite extensive work-up, including genomic testing, the underlying aetiology of polymicrogyria often remains unknown.

In a substantial proportion of patients, polymicrogyria has a genetic aetiology. Various CNVs, in particular, 22q11.2 and 1p36 deletions, have been linked to this condition, along with a rapidly growing number of monogenic causes, including several metabolic disorders (Supplementary Table 2). Dozens of genes implicated in different pathways or groups of related disorders, including the mTORopathies (affecting the PI3K–AKT–mTOR pathway), the tubulinopathies and the RABopathies, have been associated with polymicrogyria^121^.

A common cause of polymicrogyria is a congenital CMV infection, which is thought to account for 12–30% of cases, or even more among patients with specific white matter changes^62,64^. Congenital CMV infection should be suspected if polymicrogyria is observed in the presence of clinical features such as microcephaly and congenital sensorineural hearing loss. Imaging features suggestive of congenital CMV, besides polymicrogyria, include white matter hyperintensities and intracranial calcifications^62,64,122^. Toxoplasmosis, syphilis, varicella zoster virus and Zika virus have also been associated with polymicrogyria^27,60^. Additional non-genetic causes include vascular disruptive events during pregnancy and, according to a few reports, maternal ergotamine use^123^. Twinning is also a risk factor for polymicrogyria, particularly in the case of death of a monozygotic co-twin, and in some cases of twin-to-twin transfusion syndrome, in which the donor twin is most commonly affected^124^. The association with twinning is proposed to be related to vascular disturbance and/or hypoperfusion^125^.

Dysmorphic features, multiple congenital abnormalities, megalencephaly and microcephaly are all indicative of a genetic cause, although the latter condition can also be associated with congenital infection. Evaluation of head circumference is an essential part of the clinical work-up and could assist with variant interpretation, as several genes are specifically associated with microcephaly or megalencephaly^121^. The best-known gene associated with polymicrogyria and microcephaly is *WDR62*, and germline or somatic variants in genes encoding components of the mTOR pathway, such as *PIK3CA* and *PIK3R2*, are usually associated with megalencephaly, often with other abnormalities such as vascular skin lesions and digital anomalies^121^. Calcifications on brain imaging are indicative of fetal brain injury (dystrophic calcification). However, *COL4A1* and *COL4A2* pathogenic variants can genetically predispose to fetal vascular injuries, and the pseudo-TORCH syndrome mimics congenital infection^126,127^.

Polymicrogyria can be associated with peroxisomal disorders such as Zellweger syndrome or D-bifunctional protein deficiency, and is reported in up to 65% of patients with the latter condition^128^. A peroxisomal disorder should be suspected if a child with polymicrogyria is unusually sick for an individual with a static brain malformation, particularly in the neonatal period or early infancy. Additional abnormalities might be found, including dysmorphic features, hepatomegaly and profound hypotonia. In addition to polymicrogyria, brain MRI will usually show severe leukoencephalopathy^129^. If a peroxisomal disorder is suspected, plasma levels of very long chain fatty acids (VLCFAs) should be checked, and further investigations such as skin fibroblast enzymatic analysis or genomic testing should be initiated.

The work-up of a patient with polymicrogyria first requires astute clinical assessment and review of the brain MRI scan. If CMV is suspected, attempts should be made to retrieve the Guthrie neonatal blood spot for CMV PCR. VLCFA analysis should be requested if a peroxisomal disorder is suspected. CMA remains the first tier of genomic analysis. Although many genes have been associated with polymicrogyria, the yield of standard genomic testing is generally ~20% (unpublished work from Neuro-MIG laboratories). Deep sequencing might be required to identify mosaic variants, especially in patients with megalencephaly. However, patients with mosaic *PIK3R2* mutations and normal OFC have been reported.

#### Cobblestone malformation

All currently known COB syndromes are genetic and inherited in an autosomal recessive mode. A major group is the dystroglycanopathies, which are linked to various genes required for O-glycosylation of α-dystroglycan (Supplementary Table 1). Patients often have muscular dystrophy with markedly elevated serum creatine kinase levels. Moreover, eye involvement, such as severe myopia or structural malformations, is frequently observed. Recurrent biallelic microdeletions at the *ISPD* locus are the most common cause of dystroglycanopathies. Other COB syndromes include laminopathies, congenital disorders of glycosylation and basement membrane transmigration disorders (reviewed by Dobyns et al.^27^). At the imaging level, COB can be difficult to distinguish from polymicrogyria^27^, but creatine kinase analysis and/or an ophthalmological examination can potentially guide the clinical diagnosis^25^.

Differentiation of COB syndromes from polymicrogyria might be especially challenging on low-resolution images and at a young age when myelination is still ongoing (from 3 months to 2 years of age). Useful distinguishing characteristics include the intracortical striations that appear at regular intervals vertical and perpendicular to the grey–white matter border in COB and that differ from the chaotic striations seen in polymicrogyria^27^. Other structural malformations that can co-occur with COB include hydrocephalus, brainstem hypoplasia and cerebellar cysts. The white matter might show an abnormal MRI signal and small cysts. However, what clearly appears as polymicrogyria on MRI can present as typical neuronal overmigration on microscopic examination, suggesting that COB and polymicrogyria have a common pathogenesis^130^.

#### Tubulinopathies

Tubulinopathy is caused by heterozygous missense variants in any one of six tubulin-encoding genes, *TUBA1A*, *TUBB2A*, *TUBB2B*, *TUBB3*, *TUBB* and *TUBG1*. The variants probably exert dominant-negative effects on microtubule assembly and/or function. Although several pathogenic variants are recurrent, many patients harbour a unique variant, which can be difficult to confidently classify as pathogenic without functional studies^131^.

The tubulinopathies present with highly heterogeneous yet very recognizable patterns of brain malformations. The presence of a typical tubulinopathy pattern can be helpful in the interpretation of variants of uncertain significance (VOUS)^131^. Abnormalities of the cortex can be obvious or subtle, and the range encompasses microlissencephaly, pachygyria with a cortical thickness >10 mm, pachygyria with a 5–10 mm thick cortex (often more prominent in the perisylvian regions), polymicrogyria, dysgyria and a simplified gyral pattern^17,30,131,132^. The basal ganglia are usually dysmorphic, including an enlarged caudate and absent or diminutive anterior limb of the internal capsule (dividing the caudate from the putamen), resulting in a fused striatum that in turn gives the frontal horns of the lateral ventricles a characteristic ‘hooked’ appearance. Callosal abnormalities (partial or complete agenesis of the corpus callosum), ventriculomegaly, vermian dysplasia with ‘diagonal’ folia (folia crossing the midline at an oblique angle), cerebellar hypoplasia and asymmetric hypoplasia of the brainstem might also be seen^30,31,131,133^. *TUBB3* pathogenic variants can cause an ocular motility disorder, known as congenital fibrosis of the extra-ocular muscles type 3, with or without MCD or axonal polyneuropathy^132^.

Pathogenic variants in *DYNC1H1* and *KIF2A*, which encode microtubule-associated motor proteins, also lead to a spectrum of MCDs, ranging from pachygyria to dysgyria. Similar to the tubulinopathy spectrum, most individuals demonstrate a large caudate and vermian hypoplasia. *DYNC1H1* variants can be associated with peripheral nerve disease ranging from fetal akinesia to spinal muscular atrophy with lower extremity predominance^134^.

#### FCD and hemimegalencephaly

Somatic and/or germline variants in numerous PI3K–AKT–mTOR pathway genes, including *TSC2*, *TSC1*, *MTOR*, *PIK3CA*, *AKT3*, *RHEB*, *DEPDC5*, *NPRL3* and *NPRL2*, are known to be associated with malformations within the FCD–hemimegalencephaly spectrum^55,135–139^. TSC encompasses a wide spectrum of severity and clinical presentation, including FCD, and the diagnosis has consequences for surveillance and treatment^79^. In people who present with FCD, the skin and MRI should be checked for manifestations such as hypomelanotic macules, shagreen patch, additional FCD foci and subependymal nodules. If any of these features are present, a full diagnostic work-up including *TSC1*/*TSC2* testing is recommended^140^. Germline pathogenic variants in the GATOR1 complex genes *DEPDC5*, *NPRL2* and *NPRL3* are associated with focal onset seizures with or without FCD on imaging. In families with epilepsy in particular, these genes should be carefully checked for SNVs and CNVs that segregate in an autosomal dominant pattern with reduced penetrance^141–143^. Two-hit models involving germline plus somatic variants in *TSC2* and *DEPDC5* have been proposed to explain the aetiology of TSC-associated FCD and isolated FCD type IIA^141,142,144^. In recent years, somatic mutations in *SLC35A2*, which encodes an enzyme involved in glycosylation, have been found in focal epilepsy specimens and seem to be specific to FCD type I^137,145,146^. Analysis of resected brain tissue using deep sequencing and single-cell techniques might be required for detection of somatic mutations.

#### Cerebrovascular disorders associated with MCDs

Prenatal and postnatal cerebrovascular events can lead to ischaemic and disruptive brain malformations, including schizencephaly, polymicrogyria, intracranial calcifications, cysts and porencephaly. Disorders with a vascular and/or inflammatory basis, such as familial stroke, pseudo-TORCH syndrome, Aicardi–Goutières syndrome, leukoencephalopathy with cortical cysts, and cerebral microangiopathy syndromes with calcifications and cysts, can cause damage to the developing brain. A case series of 119 individuals with intracranial calcifications revealed a specific diagnosis in 50% of the cases^147^. Of these, 33 had Aicardi–Goutières syndrome, 6 had *OCLN-*related pseudo-TORCH syndrome and 3 had a *COL4A1-*related disease. Pathogenic variants in *USP18* have been associated with cerebral haemorrhage in utero, leading to polymicrogyria^148^. However, polymicrogyria is a rare feature in cerebrovascular disorders.

Several reports have shown porencephaly, schizencephaly, polymicrogyria and PVNH associated with *COL4A1* pathogenic variants, which cause imbalance or structural distortion of the collagen IV triple helix^126,149,150^. Evidence for a link between *COL4A2* and MCDs is weaker, although, considering the functional interactions between the two collagen IV proteins, *COL4A1* and *COL4A2* should be tested together^149^. Despite reports of *EMX2* as a ‘schizencephaly gene’, evidence of a role for *EMX2* mutations in schizencephaly is lacking^151,152^.

A list of genes that have been associated with early-onset and often severe cerebrovascular phenotypes is provided in Supplementary Table 4.

## Laboratory requirements

### Chromosomal microarray analysis

A survey within the Neuro-MIG network, which was conducted in preparation for this Consensus Statement, indicated that multiple different microarray platforms can be used, with no specific technology showing a clear advantage.

When choosing CMA platforms for MCD diagnostics, special attention should be paid to the exon-level resolution of genes in which single-exon aberrations have been described (Supplementary Table 3). Single nucleotide polymorphism arrays have the advantage of detecting regions of homozygosity, thereby facilitating diagnostics in consanguineous families. Mosaic CNVs showing as little as 15–20% chromosomal mosaicism were successfully detected in patients with neurodevelopmental disorders^153^. We anticipate that CMA will become redundant in the future as NGS costs further decrease and algorithms for CNV analysis from NGS data become more robust.

### High-throughput sequencing

As MCDs constitute a genetically heterogeneous group of disorders and the number of known disease-associated genes is rapidly increasing, we strongly recommend genome-wide testing approaches combined with targeted evaluation of genes that are currently implicated in MCDs (the ‘slice approach’). If the results of these tests are negative, the strategy can be expanded to a full trio exome analysis after appropriate genetic counselling. Neuro-MIG network laboratories are applying various exome enrichment strategies with comparable efficiency across the platforms and compliance with published NGS guidelines^154,155^. Most current exome sequencing enrichment kits provide sufficient coverage to offer an MCD panel as a type A or type B test^154^. The terms type A and type B refer to the definitions from the current guidelines for diagnostic NGS from the European Society of Human Genetics (ESHG), whereby the laboratory guarantees >99% reliable reference or variant calls of the target regions (type A) or describes exactly which regions are sequenced at >99% reliable reference or variant calls (type B)^154^.

### Variant calling and prioritization

Our experience shows that an average per base coverage of 100 reads with a minimum coverage of 30 reads is sufficient for reliable calls within coding and flanking intronic regions. Neuro-MIG network members preferentially use a variant calling threshold of 20% of the non-reference (alternative) reads and variant calling is performed within exons and 10 bp of the flanking intronic sequence (80% consensus). However, deep intronic variants affecting splicing have already been described in several MCD-associated genes (Supplementary Table 5). Such variants need to be considered in patients with highly suggestive phenotypes, but might require genome or targeted sequencing.

The described approach is applicable for the identification of constitutional (germline) and high-grade mosaic variants (>30% of cells). Special considerations regarding detection and validation of low-grade mosaic variants are summarized in the section ‘Detecting mosaic variants’ below.

Supplementary Table 2 provides a curated list of the core MCD-associated genes, including information on the observed mutational spectrum and associated phenotypes. Supplementary Table 3 summarizes selected genes associated with syndromic, often postnatal microcephaly. Microcephaly is a frequent accompanying feature of these conditions but is not a key manifestation. Genes associated with disorders that always present with microcephaly are listed in Supplementary Table 2. Taking into account the number of novel disease-associated genes that are emerging, we strongly suggest updating the gene lists according to the current literature every 6 months.

Variant interpretation follows the general recommendations of EuroGentest, the ESHG and the American College of Medical Genetics and Genomics (ACMG)^154,156^.

As all MCD entities are rare disorders, we recommend classifying a variant as benign if the allele frequency is >1% in the Genome Aggregation Database (gnomAD), which differs from the ACMG stand-alone evidence of benign impact with an allele frequency of >5%^157^. As the Neuro cohort of gnomAD includes individuals with neuropsychiatric disorders, which represent a rare manifestation of MCDs, one should consider excluding variants from this cohort when estimating gnomAD allele frequency, as pathogenic MCD-associated variants might be present. The presence of a variant as a homozygous allele in multiple (at least five) individuals in gnomAD strongly suggests its benign impact and irrelevance for the phenotype. However, one should be careful to check that the variant is truly homozygous and not hemizygous, combined with a deletion of the second allele. The impact of a homozygous SNV might differ substantially from the impact of deletion of one allele and the same SNV on the remaining allele^158^.

#### Pitfalls in variant prioritization

In-house variant databases, which contain data from a single institution, are another important source to distinguish benign from potentially causative variants. However, some MCD-relevant genes, especially those encoding tubulin, which are prone to read-alignment errors, might have high false-positive in-house frequencies. One *TUBB2B* pathogenic variant, Ala248Val^159^, was listed in gnomAD with an allele frequency of 3% but is currently flagged as failed — that is, probably an artefact — by random forest filters. However, when in-house data are analysed, this variant might erroneously show up in control samples in up to 30% of the reads (K.S., unpublished work) and might, therefore, be filtered out as a ‘frequent’ in-house variant, despite being pathogenic. On the basis of this example, we suggest that manual curation of in-house variants in the tubulin-encoding genes should include consideration of mapping quality and comparison of in-house frequencies with the curated gnomAD dataset. Sanger sequencing of *TUBB2B* could be considered in undiagnosed patients with an MCD pattern highly suggestive of a tubulinopathy. In the near future, such misalignment errors should be solved through high-resolution mapping and application of long-read DNA sequencing platforms^160^.

The presence of highly homologous pseudogenes also complicates accurate variant calling for a number of MCD-relevant target genes^161^ (Supplementary Table 2).

#### Penetrance of MCD-associated variants

With the exception of X-chromosomal genes such as *ARX* and *DCX*, and gene encoding components of the GATOR1 complex, variants in other MCD-associated genes seem to be fully penetrant, as carrier probands always show characteristic structural changes in the brain. However, individuals with these variants might be clinically asymptomatic and therefore never undergo brain MRI. In the case of inheritance of likely pathogenic variants from apparently unaffected parents, parental brain imaging is essential for accurate variant interpretation^91,110^. Female carriers of the X-chromosomal variants might be clinically unaffected and have normal brain scans^43,162^. Incomplete and/or age-related penetrance were reported for variants mainly associated with a seizure phenotype (for example, GATOR1 complex genes^163^); therefore, variants inherited from unaffected parents might be considered causative.

### Clinical laboratory report

The final laboratory report, including reporting of incidental findings, should follow the general requirements published by EuroGentest, the ESHG and the ACMG, as well as country-specific guidelines for genetic laboratory reports.

If the review board includes a medical professional with sufficient expertise in MRI interpretation, we recommend that MRI scans should be presented together with the clinical information and relevant variants. Relevant clinical information and brain imaging are important for accurate interpretation of the variants and should be actively requested.

If parents and similarly affected siblings (if applicable) were not analysed together with the index patient, segregation analysis must be strongly recommended in the final report.

All pathogenic and likely pathogenic variants (class 5 and class 4 variants, respectively) must be included in the final report. The final report should also contain all VOUS (class 3 variants) in MCD-associated genes. The laboratory should consider including protein-altering de novo, homozygous or compound heterozygous rare variants in potentially relevant genes of uncertain significance in the final report. The relevance for the MCD phenotype might be determined on the basis of the expression pattern of the gene or its potential importance for human brain evolution (human-specific genes or transcripts). Despite the fact that most MCD-associated genes are evolutionarily conserved, primate-specific genes and isoforms should not be ignored as they can be linked to neurodevelopmental disorders^164^. The relevance of such variants must be continually re-evaluated over time.

High-resolution, single-exon-level CNV analysis is essential to complement the sequencing report. CNV analysis can be provided with different methods including CNV calling from NGS data if robustly established and validated, multiplex ligation-dependent probe amplification, quantitative PCR or customized high-resolution microarrays.

The final report must specify whether CNV analysis has been performed, including information about the genes analysed and methods used for the analysis. If no copy number analysis has been carried out, the report must contain information about the genes that require copy number tests.

If (likely) pathogenic variants or VOUS have been identified, patients and/or their families should be referred to a clinical geneticist for return of results and counselling on their clinical and prognostic implications.

We recommend sharing VOUS in the available databases, such as ClinVar and the Leiden Open Variation Database. Depending on the local ethical and legal regulations, some laboratories might choose to use different country-specific databases.

## Additional considerations

### Detecting mosaic variants

Mosaic (postzygotic somatic) mutations, including mutations in PI3K–AKT–mTOR pathway genes, as well as in *DCX*, *LIS1*, *FLNA* and *TUBB2B*, have been described in a wide range of MCDs^54^. Mosaic mutation variant detection requires dedicated deep sequencing and bioinformatics tools, as these variants are likely to be missed by standard-coverage exon sequencing, especially in blood-derived DNA^58^. When available, affected brain tissue is the recommended tissue for genetic testing. If this tissue is not available, the use of ‘proxies’ such as saliva or skin-derived fibroblasts is recommended over lymphocytes when a mosaic disorder is suspected^58,165^. Ideally, multiple tissues from the same individual should be examined.

Reliable testing requires a targeted approach to specific loci, using a customized gene panel with ultra-deep sequencing (for example, >1,000-times coverage). A gene panel for PI3K–AKT–mTOR-related syndromes is provided in Supplementary Table 6. As a general rule, hybridization-based assays offer superior performance over amplicon assays^166^. However, amplicon protocols with unique molecular identifiers during library preparation have also proved effective for detecting somatic mutations^167^. The variant-calling algorithm (percentage of non-reference allele reads) must be adapted for detecting low-grade mosaic SNVs.

As false-positive mosaic mutation calls can arise from many different sources, we strongly suggest confirmation of every low-grade mosaic variant using an orthogonal technology such as droplet digital PCR or a second independent round of ultra-deep sequencing^168–170^.

### Neuropathological work-up

Detailed neuropathological examination, biobanking and genetic testing are required after epilepsy surgery or autopsy in patients with MCDs, and also after sudden unexpected death in epilepsy, as individuals who die as a result of sudden unexpected death in epilepsy might have a previously undiagnosed MCD.

In 2016, the task force of neuropathology from the International League Against Epilepsy (ILAE) Commission on Diagnostic Methods published a consensus standard operational procedure for collection and processing of cortical samples from patients with MCDs such as FCDs^94^. Whenever feasible, anatomically intact surgical neocortical samples should be obtained to allow systematic analysis to identify the affected area. Correct orientation of the cortical sample and determination of its relationship to neurophysiologically aberrant sites and MRI findings requires an interdisciplinary diagnostic approach with good communication between pathology, neurology, radiology and neurosurgical teams. Representative tissue should be apportioned for histology and biobanking. Brain tissue-derived DNA is often required for genetic diagnosis in FCD and hemimegalencephaly; thus, highly standardized tissue processing is recommended. A neuropathologist should be involved in the interpretation of the brain pathology, and molecular biologists (or pathologists) and geneticists should participate in the set-up and analysis of the sequencing results^171^. A consensus protocol with details of how to best process resected brain specimens for somatic mutational analysis to detect mosaic variants for hemimegalencephaly, FCD types I and II, and other MCDs is under development by a task force of the ILAE (E.A., unpublished work).

Analysis of low-level mosaic mutations, such as those reported in FCDs^57,136,142,172^, requires careful selection of brain regions and cells to ensure enrichment of the mutated cells, followed by deep sequencing^136,138^. A study published in 2019 used resected brain tissue from a large cohort of patients after epilepsy surgery to explore the possibility of detecting low-level somatic mutations in unmatched formalin-fixed paraffin-embedded (FFPE) brain tissue samples (that is, brain samples without a blood sample from the same patient). FFPE samples often represent the most relevant samples in the standard neuropathological diagnostic approach to MCDs^146,173^. The researchers showed that deep sequencing, even when applied to unmatched FFPE brain tissues, can be used to accurately and efficiently detect low-level somatic mutations.

## Conclusions

In this Consensus Statement, we present a diagnostic work-up for individuals affected by brain malformations within the MCD spectrum, encompassing current best practices and recommendations based on the consensus of a multidisciplinary group of international experts within the Neuro-MIG network. With this approach, we aim to increase diagnostic yield, thereby improving patient care and management worldwide and facilitating the development of targeted therapeutic approaches in the long term.

## Supplementary information

Supplementary Table

Supplementary Box
