# Supplementary material for: International consensus recommendations on the diagnostic work-up for malformations of cortical development
Source: Nat Rev Neurol. 2020 Sep 7;16(11):618–35. doi: 10.1038/s41582-020-0395-6 (PMC7790753; doi:10.1038/s41582-020-0395-6)
Supplement: Supplementary file 1 — Supplementary Table [file 41582_2020_395_MOESM1_ESM.pdf]

nature research
https://doi.org/10.1038/s41582-020-0395-6

Supplementary information

International consensus recommendations on the diagnostic work-up for malformations of cortical development

In the format provided by the authors and unedited

Supplementary Table 1 | Delphi consensus, diagnostic work-up for malformations of cortical development

Statement #	Percentage consensus	Statement
**1**	>90%	Malformations of cortical development (MCDs) uniformly describe a heterogeneous group of disorders resulting from abnormal development of the cerebral cortex
**2**	>90%	Altogether MCDs represent a major cause of intellectual disability (ID), autism, epilepsy and cerebral palsy
**3**	>90%	MCDs are frequently genetic, but can also have environmental or multi-factorial origins
**4**	>90%	We define megalencephaly (MEG) as an abnormally large brain that exceeds the mean for age and gender by ≥2 SD. MEG specifically refers to a brain size that is ≥2 SD above the mean and is primarily a developmental brain disorder, whereas macrocephaly (defined as an occipitofrontal circumference (OFC) ≥2 SD above the mean) has a wide variety of causes besides MEG, including ventriculomegaly, hydrocephalus and skeletal dysplasia
**5**	>90%	We stress that macrocephaly between 2 and 3 SD might not represent a pathological condition. The decision whether an individual with OFC 2-3 SD requires further diagnostics must be done in the clinical context taking into account presence or absence of the developmental delay/ID, autism, epilepsy or other anomalies
**6**	>90%	Periventricular nodular heterotopia (PNH) is described as cortex-isointense nodules along the ventricular walls protruding into the lumen
**7**	>90%	Subcortical heterotopia (SUBH) is defined as grey matter located regionally in the deep cerebral white matter. This term describes a heterogeneous group of malformations and is separated from subcortical band heterotopia defined within the lissencephaly spectrum
**8**	>90%	Lissencephaly spectrum includes agyria, pachygyria, and subcortical band heterotopia. Agyria/pachygyria describes abnormal gyral pattern with absent or unusually wide gyri in combination with an abnormally thick cortical ribbon. Subcortical band heterotopia is a description of a smooth band of neurons separated from the cortex by a few millimetres of white matter
**9**	>90%	Cobblestone malformation (COB) defines an undersulcated cerebral surface, with moderately thick cortex, and jagged grey-white matter border with frequent vertical (perpendicular to the grey-white matter border) striations
**10**	>90%	Polymicrogyria (PMG) describes an excessive number of abnormally small cerebral gyri and is diagnosed by the regions of abnormal cortex with the irregular, "pebbled' cortical surface and a "stippled" grey-white matter boundary
**11**	>90%	Schizencephaly (SCH) describes a cerebral cleft lined with grey matter which extends across the full thickness of the cerebral hemispheres from the ventricular surface (ependyma) to the periphery (pial surface) of the brain
**12**	>90%	Focal cortical dysplasia (FCD) defines cortical dyslamination, with or without cytoarchitectural lesions and underlying abnormalities of white matter visible on imaging as increased cortical thickness, blurring of the cortical–white matter junction, increased signal on T2-weighted images, a radially oriented linear or conical transmantle stripe of T2 hyperintensity, cortical thinning, and/or localized brain atrophy
**13**	>90%	The term dysgyria was introduced as a transient definition of the currently poorly defined cortical malformation described as a cortex of normal thickness and grey-white boundary but with an abnormal gyral pattern characterized by irregularities of sulcal depth and/or orientation. We suggest that this term will be replaced as soon as more information about the aetiology and pathology of this type of MCD will be available
**14**	>90%	Several MCD patterns are very distinctive and have a strong causal association with a single or few disease genes
**15**	>90%	Several MCD patterns are genetically heterogeneous but are associated with specific gene families (e.g. the tubulinopathies)
**16**	>90%	The recognition of the specific MCD patterns by neuroimaging allows more targeted testing
**17**	>90%	An effort should be made to reach an aetiological diagnosis in every individual affected by MCD
**18**	>90%	The suggested strategy is not recommended for individuals with a known molecular cause of MCD that fully explains the phenotype
**19**	>90%	The suggested strategy is not recommended for individuals with a known prenatal or perinatal cause that fully explains the phenotype
**20**	>90%	The suggested diagnostic strategy is not recommended for individuals with a single periventricular heterotopic nodule without ID, seizures and/or major and/or minor anomalies
**21**	>90%	Besides complete pedigree analysis, recommended preliminary assessments include detailed prenatal and perinatal history with emphasis on placental

		insufficiency, twinning, teratogenic exposures, and documentation of the Apgar scores
**22**	>90%	Physical examination of MCD patients should include measurements of OFC, height and weight, full neurological (including muscular and peripheral nerve) and dysmorphology examination
**23**	>90%	PCR screening of archived newborn dried blood spots for suspected cytomegalovirus (CMV) infection should precede genetic testing unless a specific MCD form that is not associated with newborn infection can be reliably diagnosed
**24**	>90%	Ideally all the requests for genetic tests in MCD patients should be accompanied by a copy of the MRI scan and/or pathology report
**25**	>90%	The laboratory case review board should include a medical professional with sufficient expertise in MRI interpretation
**26**	>90%	The general MCD diagnostic pipeline includes two steps that are followed by a detailed phenotypic re-evaluation: (1) copy number variation (CNV) analysis by chromosomal microarray analysis (CMA) and (2) high-throughput sequencing (whole-exome enrichment or whole-genome sequencing (WGS)) with targeted evaluation of the extended MCD gene panel
**27**	>90%	Several MCD genes (e.g. *FLNA*, *PAFAH1B1*, *DCX*) frequently harbour intragenic deletions or duplications, some of which can be identified by standard microarrays
**28**	>90%	MCDs have been reported in a wide range of patients with abnormalities detected by CMA
**29**	>90%	We anticipate that CMA will become redundant in the future as next-generation sequencing (NGS) costs will go down and algorithms for CNV analysis from NGS data become more robust
**30**	>90%	We strongly recommend genome-wide testing approaches with the targeted evaluation of the subset of the currently known disease genes
**31**	>90%	If the exome slice approach did not result in identification of the disease cause, the analysis should be expanded with a full trio-based exome analysis after appropriate genetic counselling
**32**	>90%	The proper interpretation of both positive and negative results requires a detailed phenotype analysis, including re-evaluation of the brain MRI, to answer the question of whether the discovered mutation/CNV correlates with and fully explains the phenotype
**33**	>90%	In the case of negative results, the re-evaluation should help to decide whether the malformation really falls into the MCD group, whether additional diagnostic test might be helpful (e.g. deep sequencing or analysis of a different tissue) and whether a non-genetic cause is a more likely explanation
**34**	>90%	Patients with an MCD pattern highly specific for one or a few genes benefit from manual inspection of NGS reads and/or alternative targeted sequencing methods such as Sanger sequencing completed by the deletion/duplication testing of genes of interest
**35**	>90%	Some patients might benefit from repeat brain imaging especially if the first MRI was performed during the first weeks of life on a prematurely born child or during the active myelination period (3-18 months)
**36**	>90%	Undiagnosed patients from consanguineous pedigrees and families with multiple affected siblings require a single nucleotide polymorphism (SNP) microarray analysis to identify regions of homozygosity. If a homozygous region contains a known MCD gene compatible with the phenotype, special caution must be given to the deep intronic variants
**37**	>90%	Metabolic investigations should be considered in undiagnosed patients with PMG or COB, as a broad range of metabolic diseases manifest with cortical malformations resembling PMG and COB
**38**	>90%	Undiagnosed patients should receive trio-based WGS, preferentially within a large collaborative research network allowing rapid discovery of the novel causative genes or non-coding variants in regulatory elements
**39**	>90%	NGS with an average per base coverage of 100 reads with a minimal per base coverage of 30 reads is sufficient for reliable calls within coding and flanking intronic regions
**40**	>90%	An average per base coverage of 100 reads with a variant calling threshold of 20% is applicable for the identification of germline, constitutional and high-grade mosaic variants (>30% of the cells)
**41**	>90%	Special attention must be given to genes with single exon aberrations have already been reported as disease-causing changes. High-resolution, single-exon-level CNV analysis is mandatory to complement the sequencing report
**42**	>90%	Variant interpretation follows general recommendations of EuroGentest, the European Society of Human Genetics and the American College of Medical

		Genetics and Genomics (ACMG)
**43**	>90%	As all MCD entities are rare disorders we recommend classifying a variant as benign if the allele frequency is >1% in gnomAD; this differs from the ACMG stand-alone evidence of benign impact with an allele frequency of >5%
**44**	>90%	The presence of a variant as a homozygous allele observed in multiple (more than five) individuals in gnomAD strongly suggest its benign impact and irrelevance for the phenotype
**45**	>90%	When performing whole-exome analysis, we recommend including variants of unknown significance (VOUS) as well as protein altering de novo, homozygous or compound heterozygous rare variants in the potentially relevant genes of unknown significance (GUS) in the final report
**46**	>90%	The potential relevance for the MCD phenotype might be supported based on the expression pattern of the gene. The potentially relevant GUS should be expressed in the brain and especially in the fetal brain
**47**	>90%	We recommend to share VOUS in the available databases as well as to share information about the variants in the potential GUS, providing also the key phenotypic features
**48**	>90%	The information about the population allele frequencies as well as variant frequencies in in-house databases is essential for accurate filtering and variant interpretation
**49**	>90%	All pathogenic and likely pathogenic variants (class 5 and class 4 variants) must be included in the final report. The final report should also contain all (class 3 variants) in all genes on the MCD panel, especially VOUS in genes causing recessive MCDs even if the VOUS is heterozygous and no other variants could be identified on the second allele
**50**	>90%	The final report must specify whether CNV analysis has been performed, including information about the gene(s) analysed and method(s) used for the analysis. If no CNV analysis has been carried out, the report must contain information about the genes requiring copy number tests
**51**	>90%	Somatic (i.e. postzygotic) mutations have been described in a wide range of MCDs including mutations of the PI3K-AKT-MTOR pathway genes in FCD type 2b, hemimegalencephaly and MEG
**52**	>90%	Somatic mutations are likely missed by standard-coverage whole-exome sequencing (WES; e.g. 80-100X coverage WES), especially in blood-derived DNA, warranting high-depth NGS methods (>1000× coverage ideally)
**53**	>90%	Affected brain tissue obtained after epilepsy surgery, where available, is the recommended tissue for genetic testing for somatic mosaic mutations. If this is not available, the use of saliva or skin-derived fibroblasts is recommended
**54**	>90%	Reliable testing for mosaic mutations requires restriction to specific loci and design of a customized panel of the relevant genes (e.g., using targeted hybridization or amplicon sequencing approaches) with deep sequencing (e.g. >5000X coverage)
**55**	>90%	Because false-positive mosaic mutations calls can arise from many different sources, we recommend confirmation of every low-grade mosaic variant using orthogonal technology
**56**	>90%	Ideally, multiple tissues from the same individual should be examined to confirm the somatic nature of a mutation
**57**	>90%	Microcephaly is a highly heterogeneous brain growth disorder that can be isolated (typically with a simplified gyral pattern) or complex with additional brain abnormalities (e.g. cortical malformations, cerebellar abnormalities)
**58**	>90%	Large gene panel or exome sequencing is a recommended testing strategy in patients with microcephaly given its genetic heterogeneity
**59**	>90%	Genetic test in patients with microcephaly should preferably be pursued after exclusion of non-genetic causes, paying special attention to TORCH infections during pregnancy (toxoplasmosis, others (e.g. syphilis, varicella zoster, parvovorus B-19), rubella, CMV and herpesvirus) and the emerging Zika virus
**60**	>90%	Four distinct lissencephaly patterns are highly specific for mutations in one or two genes with diagnostic yield above 90%: (1) diffuse agyria with cortical thickness >10 mm (genes *LIS1* and *DCX*), (2) occipital agyria combined with frontal pachygyria (*LIS1*, p.402 in *TUBA1A*), (3) temporal predominant pachygyria with a cortical thickness of 5-10 mm in combination with the complete agenesis of the corpus callosum and severe hypomyelination (*ARX*) and (4) diffuse thick subcortical band heterotopia (*DCX*)
**61**	>90%	Tubulinopathies present with three types of MCD: (1) lissencephaly, including pachygyria with cortical thickness > 10mm and also 5-10 mm with variable gradient that is often more prominent in the perisylvian regions, (2) tubulinopathy-related dysgyria, and (3) simplified gyral pattern

**62**	>90%	In tubulinopathies MCDs are almost always associated with one or more non-cortical malformations including dysmorphic basal ganglia, callosal abnormalities, vermian dysplasia cerebellar hypoplasia, and asymmetric hypoplasia of the brainstem
**63**	>90%	When a tubulinopathy pattern is reliably recognized on imaging, one can pursue NGS without performing CNV analysis first. However, negative test results always require a phenotypic re-evaluation to confirm the tubulinopathy-specific MCD pattern and determine the further diagnostic strategy
**64**	>90%	All currently known syndromes associated with COB are genetic and inherited in an autosomal recessive fashion
**65**	>90%	On imaging cobblestone malformation can be confused with PMG
**66**	>90%	The diagnostic yield of the NGS gene panel is higher than that of CMA and could be considered as a first step in the diagnostic process in patients with COB in the case of a reliable radiological diagnosis
**67**	>90%	PMG is one of most frequent types of MCD and certainly one of the most heterogeneous in aetiology
**68**	>90%	PMG might be difficult to differentiate from COB, dysgyria and even pachygyria. The definite radiological diagnosis requires high quality MRI to visualize microgyri and microsulci as well as stippling of the grey-white matter junction
**69**	>90%	The first diagnostic step in patients with PMG is exclusion of congenital infections and a careful pregnancy anamnesis regarding twinning and disruptive events such as near miscarriage and trauma, followed by CMA
**70**	>90%	The yield of NGS in PMG is highest in patients with macrocephaly (~60%), lower in patients with microcephaly (~25%) and lowest in patients with normocephaly (~10%)
**71**	>90%	In patients with perisylvian PMG and negative results after general diagnostic work-up, deep sequencing of *PIK3R2* in a second tissue should be considered
**72**	>90%	In females with periventricular nodular heterotopia (PVNH) presenting as bilateral clusters of confluent nodules extend along the walls of the frontocentral lateral ventricles, *FLNA* testing can be performed first. This testing should include deletion/duplication analysis
**73**	>90%	For patients with variable presentation of PVNH not suggestive for *FLNA* mutations, the general MCD workflow with CMA and gene panel testing is the recommended approach
**74**	>90%	In individuals with one or two single nodules, normal cognitive functioning and no other congenital abnormalities, the yield of genetic testing is low
**75**	>90%	Several rare, mostly symmetrical bilateral forms of subcortical heterotopia are genetic, e.g. the mesial parasagittal form was seen in Chudley-McCullough syndrome resulting from homozygous variants in *GPSM2*, and ribbon-like heterotopia in combination with agenesis of the corpus callosum and macrocephaly seen with homozygous *EML1* mutations
**76**	>90%	For large curvilinear heterotopias, which are usually giant and asymmetric, no genetic cause is known and a vascular disruptive cause has been suggested
**77**	>90%	Cerebrovascular events during pregnancy can lead to SCH, PMG, intracerebral calcifications, cysts, porencephaly and other ischaemic and disruptive non-cortical brain malformations
**78**	>90%	Monozygotic twinning, several teratogens, young parental age and traumatic injury predispose to vascular disruption
**79**	>90%	Evidence for a role of *EMX2* mutations as a cause of SCH is low and testing of *EMX2* is not recommended
**80**	>90%	Cerebrovascular disorders associated with familial stroke, pseudo-TORCH syndrome, Aicardi-Goutieres syndrome, leukoencephalopathy with cortical cysts, and cerebral microangiopathy syndromes with calcifications and cysts may be responsible for insults to the developing brain and lead to cortical malformations, e.g. microcephaly and PMG
**81**	>90%	*COL4A1* and *COL4A2* mutations have been associated with SCH, porencephaly and polymicrogyria.
**82**	>90%	Considering the large number of rare genetic cause of early-onset cerebral vascular disorders and insufficient knowledge of the clinical spectrum, in individuals with an unexplained microcephaly or PMG/SCH and suggestive evidence for a vascular pathogenesis (e.g. calcifications, bleeding, porencephaly and associated white matter lesions), we recommend testing 27 genes currently associated with early-onset cerebral vascular and inflammatory disorders, followed by a trio WES/WGS
**83**	>90%	Postmortem pathological examination of deceased patients with MCDs may guide a targeted analysis of the most probable causative genes and contribute to the interpretation of the pathogenicity of genetic variants identified through NGS

**84**	>90%	Availability of brain tissue is essential to identify somatic mutations in affected regions in patients with FCD
**85**	>90%	A neuropathologist with expertise in developmental neuropathology should be involved in the interpretation of the brain pathology and the selection of regions and cells for analysis of somatic mutations to increase diagnostic yield
**86**	>90%	A neuropathological evaluation using a panel histochemical stains and antibody immunoreactivities recommended by the International League Against Epilepsy classification system is essential for the correct diagnosis of FCD type
**87**	>90%	Whenever feasible, anatomically intact surgical neocortical samples obtained during epilepsy surgery are desirable to allow a systematic analysis with identification of the affected area. Representative tissue should be apportioned for histology and bio-banking (snap frozen, formalin or paraformaldehyde fixed etc.)
**88**	>90%	Somatic, germline and germline together with somatic pathogenic variants (second hit model) of a growing number of PI3K-AKT-MTOR pathway genes are known to be associated with FCD including, in order of frequency, *TSC2*, *TSC1*, *MTOR*, *PIK3CA*, *DEPDC5*, *NRPL3* and *NPRL2*
**89**	>90%	Germline mutations in GATOR1 complex genes, including *DEPDC5*, *NPRL2* and *NPRL3*, can cause FCD in a minority of patients
**90**	80%-90%	Microcephaly is confusingly defined in the literature; in our recommendations we use the term microcephaly to describe congenital microcephaly as a reduction in the OFC by 3 SD compared with age and gender matched controls at birth. Postnatal microcephaly is defined as OFC 2 SD below age and gender matched controls
**91**	>90%	MCD being diffuse or focal, bilateral or unilateral does not reliably distinguish genetic from non-genetic causes
**92**	>90%	The single criterion to apply a suggested diagnostic approach is a diagnosis of MCD based on neuroimaging or neuropathology or the presence of primary microcephaly. However, in people with primary microcephaly, pretest neuroimaging is strongly recommended
**93**	>90%	Genetic testing should be considered in all individuals presenting with MCD due to unexplained cerebrovascular disease
**94**	80%-90%	A clinical genetic evaluation with family counselling should take place prior to any genetic testing in MCD patients
**95**	70%-75%	A comprehensive phenotype analysis always remains an important part of the diagnostic workflow but it might be conducted after molecular testing
**96**	75%-80%	If not performed previously, consider karyotype in undiagnosed MCD patients
**97**	80%-90%	A variant calling threshold of 20% of the aberrant reads is preferentially used for germline variants, with the variant calling being performed within exons and 10 bp of the flanking intronic sequence
**98**	>90%	Clinical reports must be reviewed in an interdisciplinary manner. If the review board includes medical professionals with sufficient expertise in MRI interpretation, MRI scans must be presented together with the clinical information and relevant variants. Relevant clinical information and brain imaging are crucial for an accurate report and must be requested if not provided or insufficient at the time of the test order
**99**	75%-80%	All genetic results must be communicated with the patients and/or families by medical geneticists
**100**	>90%	Testing in patients with MEG as a part of the PI3K-AKT-MTOR-associated spectrum of disorders is ideally performed on affected (lesion) tissues such as brain, rather than peripheral samples (such as blood), using deep sequencing approaches
**101**	80%-90%	SNP array is a preferential CNV test method in patients with COB since it also provides homozygosity mapping, pointing to a potential disease locus

Supplementary Table 4 | Cerebrovascular MCD-associated genes

Gene	Syndrome	Inheritance
ACTA2	Multisystemic smooth muscle dysfunction syndrome	AD
ADAR1	Aicardi-Goutieres syndrome	AR
COL4A1	Small-vessel brain disease	AD
COL4A2	Small-vessel brain disease	AD
CSF1R	Brain abnormalities, neurodegeneration, and dysosteosclerosis	AR
CTC1	Cerebroretinal microangiopathy with calcifications and cysts	AR
DENND5A	Epileptic encephalopathy	AR
FAM20C	Raine syndrome	AR
FLVCR2	Fowler syndrome	AR
GNAQ	Sturge-Weber syndrome	Mosaic
GPHN	Molybdenum cofactor deficiency	AR
IBA57	Multiple mitochondrial dysfunctions syndrome	AR
IFIH1	Aicardi-Goutieres syndrome	AD
ISG15	Immunodeficiency 38	AR
JAM3	Hemorrhagic destruction of the brain, subependymal calcification, and cataracts	AR
MOCS1	Molybdenum cofactor deficiency	AR
MOCS2	Molybdenum cofactor deficiency	AR
OCCL	Pseudo-TORCH syndrome	AR
PCDH12	Aicardi-Goutieres-like syndrome	AR
POLR3B	Leukodystrophy	AR
RANBP2	Infection-induced necrotising encephalitis	AD
RARS2	Pontocerebellar hypoplasia, type 6	AR
RNASEH2A	Aicardi-Goutieres syndrome	AR
RNASEH2B	Aicardi-Goutieres syndrome	AR
RNASEH2C	Aicardi-Goutieres syndrome	AR
RNASET2	Cystic Leukoencephalopathy	AR
SAMHD1	Aicardi-Goutieres syndrome	AR
SNORD118	Labrune syndrome	AR
TINF2	Dyskeratosis congenita, Revesz syndrome	AD
TREX1	Aicardi-Goutieres syndrome	AR
USP18	Pseudo-TORCH syndrome	AR

AD, autosomal dominant; AR, autosomal recessive, MCD, malformation of cortical development.

Supplementary Table 5 | Intronic disease-causing variants in the MCD-associated genes

Gene	Genomic coordinates [hg19]	Reference allele	Alternative allele	Zygosity	HGVS nomenclature	Effect on the protein level	Phenotype	Reference
ARX	ChrX:25033657	T	C	Hemizygous	NM_139058.2:c.196+2T>C	Skipping of exon 1	Lissencephaly, with abnormal genitalia	PMID: 14722918
ARX	ChrX: 25028376	G	C	Hemizygous	NM_139058.2:c.1119+1G>C	Skipping of exon 3	Lissencephaly, with abnormal genitalia	PMID: 14722918
ATRX	ChrX:76874459	A	T	Hemizygous	NM_000489.3:c.5273-10T>A	Skipping of exon 9	Microcephaly in alpha-thalassaemia/mental retardation syndrome	PMID: 8644709
CDK5RAP2	Chr9:123182253	T	C	Homozygous	NM_018249.5:c.4005-15A>G	Insertion of a new splice acceptor site	Microcephaly, primary, autosomal recessive	PMID: 15793586
CHMP1A	Chr16:89718067	C	A	Homozygous	NM_002768.4:c.28-13G>A	Aberrant splice acceptor site	Microcephaly in pontocerebellar hypoplasia type 8	PMID: 23023333
FKTN	Chr9:108401937_108401938insAB185332.1	T	ins	Homozygous	NM_006731.2:c.*4392_*4393insAB185332.1	Insertion of a 3,062 bp transposon in the 3-prime UTR resulting in loss of transcription of *FKTN* mRNA	Cobblestone malformation in Fukuyama congenital muscular dystrophy	PMID: 9690476
FKTN	Chr9:108368857	G	T	Compound heterozygous with 3 kb insertion	NM_006731.2:c.647+2084G>T	Pseudoexon between exon 5 and exon 6	Cobblestone malformation in Fukuyama congenital muscular dystrophy	PMID: 28680109
MLC1	Chr22:50502853	A	C	Homozygous	NM_015166.3:c.895-226T>G	Insertion of one pseudoexon of 246 bp in intron 10, which contains in frame stop codons	Macrocephaly in megalencephalic leukoencephalopathy with subcortical cyst	PMID: 22552818
PLK4	Chr4:128819589	C	G	Homozygous	NM_014264.4:c.2811-5C>G	Cryptic	Microcephaly and	PMID: 25344692

						splice acceptor activation	chorioretinopathy	
RARS2	Chr6:88244587	G	A	Compound heterozygous with c.1A>G, p.?	NM_020320.3:c.613-3927C>T	Skipping of exons 6-8	Microcephaly in pontocerebellar hypoplasia type 6	PMID: 26083569
RARS2	Chr6:88279230	T	C	Homozygous	NM_020320.3:c.110+5A>G	Skipping of exon 2 with the subsequent frameshift	Microcephaly in pontocerebellar hypoplasia type 6	PMID: 17847012
RBBP8	Chr18:20581745	T	G	Homozygous	NM_002894.2:c.2347+53T>G	Exon 15 donor splice site skipped	Primary autosomal recessive microcephaly and Seckel syndrome spectrum	PMID: 21998596
RTTN	Chr18:67802412	A	G	Homozygous	NM_173630.3:c.2885+8A>G, P.?	Cryptic donor site with premature stop	Severe microcephaly facial dysmorphism	PMID: 26608784
RTTN	Chr18:67801710	A	G	Homozygous	NM_173630.3:c.2953 A>G p.Arg985Gly	Exon skipping	Microcephaly, failure to thrive, developmental delay	PMID: 26940245
RTTN	Chr18: 67815044	G	A	Homozygous	NM_173630.3:c.2309+1093G>A	New splice acceptor	Primary microcephaly, intellectual disability	PMID: 30879067
RTTN	Chr18: 67727297	T	A	Heterozygous	NM_173630.3:c. 4748–19T>A	Novel splice acceptor	Intrauterine growth restriction, microcephaly	PMID: 30879067

HGVS, Human Genome Variation Society. MCD, malformation of cortical development.

Supplementary Table 6 | MCD-associated genes with mosaic mutations

Gene	Type(s) of MCD	Mutation type	References (PMID)	Hot spots
*AKT1**	Megalencephaly	Missense	21793738	p.Glu17Lys
*AKT3**	Megalencephaly	Missense	28969385, 25091978, 23794269	p.Glu17Lys
DCX	Lissencephaly, heterotopia	Missense, splicing, large deletions	25140959, 22833188	
*DEPDC5**	Focal cortical dysplasia	Missense	25623524	
FLNA	Heterotopia	Frameshift, splicing	25140959, 15459826	
*MTOR**	Megalencephaly, polymicrogyria and focal cortical dysplasia	Missense	27159400, 29281825, 27830187	p.Ser2215
*NPRL2**	Focal cortical dysplasia	Stop	29281825	
PAFAH1B1 (*LIS1*)	Lissencephaly, heterotopia	Stop, missense, splicing	25140959	
*PIK3CA**	Megalencephaly, polymicrogyria	Missense	27631024"	p.Gly914Arg, p.Met1043Ile
*PIK3R2**	Megalencephaly, polymicrogyria	Missense	26520804	p.Gly373Arg
*TSC1**	Focal cortical dysplasia	Stop, frameshift, splicing	26540169, 17287951	
*TSC2**	Focal cortical dysplasia	Stop, frameshift, splicing	26540169, 17287951	
TUBB2B	Lissencephaly, heterotopia	Missense	25140959	

*Genes recommended being included in deep sequencing panel in patients with features of PI3K-AKT-mTOR-related syndromes or focal cortical dysplasia. MCD, malformation of cortical development.

Supplementary Box 1 | Case reports demonstrating how phenotype evaluation influences interpretation of the molecular test results

Case report 1 | "Double-trouble": complex phenotype due to two pathogenic mutations

A boy was born as a second child of healthy unrelated parents. He showed severe muscular hypotonia, global developmental delay, and severe microcephaly of –4.3 SD at the age of 2 years. His brain MRI demonstrated diffuse agyria with cortical thickness 10-20 mm and enlarged ventricles and enlarged axial spaces (Figure 1). The condition was evaluated as microlissencephaly and trio-based exome sequencing was requested with the evaluation of malformation of cortical development (MCD)-associated genes as a first diagnostic step. No pathogenic or likely pathogenic variants were found in the MCD panel. An open exome evaluation revealed a de novo missense variant in ARID2 (NM_152641.2:c.4523G>A:p.Gly1508Asp. chr12[hg19]:g.46246429G>A) fulfilling the American College of Medical Genetics and Genomics (ACMG) criteria for the likely pathogenic mutation (criteria PS2, PM2, PP3 and BP1)[1]. Heterozygous loss-of-function mutations in *ARID2* are associated with the Coffin-Siris syndrome or Coffin-Siris like phenotype.[2,3] Although severe microcephaly has not been reported in patients with *ARID2* mutations, microcephaly is a feature of the Coffin-Siris syndrome due to *SMARCE1* mutations.[4] In contrast, lissencephaly, especially in the form of diffuse agyria, has never been associated with any mutations in the genes encoding the SWI/SNF complex and therefore cannot be explained by a de novo variant in *ARID2*. Copy number analysis on next-generation sequencing (NGS) data followed by chromosomal microarray testing revealed a deletion of *PAFAH1B1* with the adjacent genes compatible with the observed cortical phenotype. Although postnatal microcephaly is a frequent feature of *PAFAH1B1*-associated lissencephaly, it usually remains in the mild-to-moderate range in contrast to severe congenital microcephaly observed in the reported patient.[5] Such an unusual presentation is most probably due to the presence of two independent genetic changes. A careful MRI review prompted an additional copy number variation analysis even after one likely pathogenic variant partly compatible with the phenotype was identified.

Figure 1

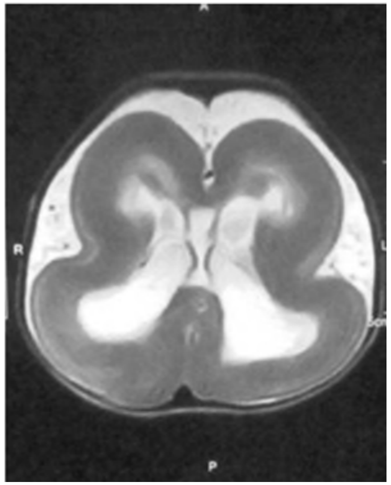

Case report 2 | Expansion of the phenotypic spectrum

A girl presented at birth with hydrocephalus, respiratory distress and severe feeding difficulties. Birth growth parameters were within the normal range. Tonic seizures started at 3 weeks of age and developed into generalized tonic-clonic seizures that were refractory to therapy. She required feeding via G-tube. At the age of 3 years she showed profound developmental disability with absent speech and no independent sitting. Height and head circumference were normal (P36 and P10 respectively). She also demonstrated astigmatism, sensory exotropia, and cortical visual impairment, sleep disordered breathing, neurogenic bladder, and chronic constipation. The girl had multiple facial minor anomalies such as deep set eyes, roving eye movements, flat facies, and tented upper lip with downturned corners of the mouth. Brain MRI showed diffuse bilateral polymicrogyria, enlarged lateral ventricles, severe dysplasia of the basal ganglia, enlarged tectum and mild cerebellar hypoplasia (Fig. 1 in Platzer et al. [6]). This malformation pattern was evaluated as suggestive for tubulinopathy. Chromosomal microarray findings were normal and open trio-exome sequencing revealed no relevant variants in the MCD panel genes but uncovered a de novo heterozygous missense variant in *GRIN2B*: NM_000834.3:c.1916C>T p.(Ala639Val). This variant was classified as a likely pathogenic mutation according to ACMG criteria (PS2, PM2, PP2 and PP3). Mutations in *GRIN2B* were described in patients with West syndrome, intellectual disability with or without seizures and epileptic encephalopathies.[7] None of the mutations in the genes encoding N-methyl-D-aspartate receptor subunits have been associated with malformations of cortical development at the time of the initial evaluation and therefore an additional cause was suggested for the complex MCD phenotype. No additional relevant variants could be identified after re-analysis of the trio-whole-exome sequencing (WES) data including manual inspection of the reads for tubulin-encoding genes. Referral of additional patients presenting with the overlapping MCD pattern and de novo missense variants in *GRIN2B* led to re-evaluation of the original report with confirmation of the single diagnosis of *GRIN2B* encephalopathy as well as expansion of the phenotypic consequences of channelopathies.[6]

Case report 3 | Distinct gene-specific phenotype*

A girl was born at term with normal growth parameters and presented with seizures at the age of 2 weeks that were subsequently well controlled. She did not show any other major or minor anomalies. Brain MRI at the age of 1.5 months showed distinct pattern of frontal lissencephaly with a cortical thickness of 7-10 mm combined with the severe cerebellar hypoplasia and hippocampal dysplasia (Figure 2). Following a radiological diagnosis of lissencephaly, the child underwent genome-wide chromosomal microarray and WES analysis. Chromosomal microarray findings were normal. Sequencing results reported two variants of unknown significance in *RELN* — NM_005045.3:c.[5200C>G]?[8489+4_8489+7delAGTA] (p.[(Leu1734Val)]?[-]) — that were confirmed to be inherited from the father and as two variants located on the same allele could not alone be responsible for the phenotype. Expert MRI review confirmed the radiological diagnosis of lissencephaly specifying that the MCD pattern especially in combination with the severe cerebellar and hippocampal dysplasia is typically associated with mutations in *RELN* or its receptor VLDLR (PMID: 28440899). The clinical diagnosis of reelinopathy with detection of one abnormal *RELN* allele prompted copy number analysis of *RELN* using multiplex ligation-dependent probe amplification, which confirmed the presence of a deletion of exon 4 inherited from the mother. The recognition of the distinct malformation pattern facilitated the detection of the second causative mutation, enabling the family to be provided with a molecular diagnosis.

Figure 2

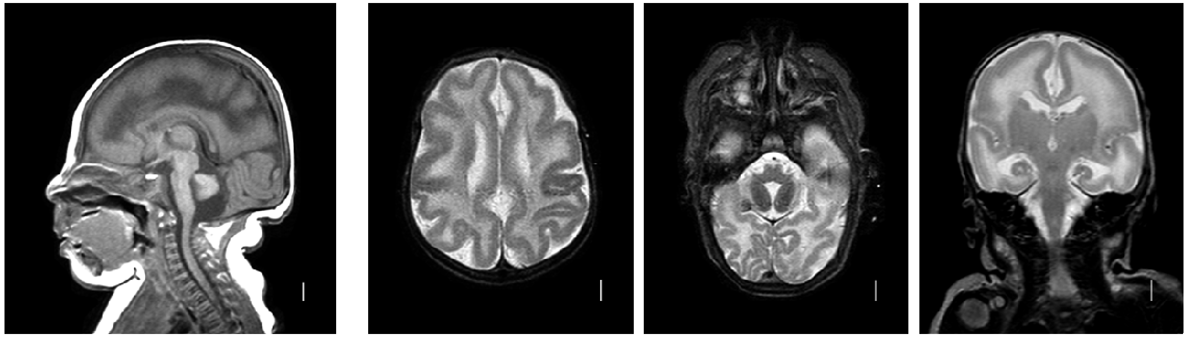

*Case published previously as individual LR14-063[8].

References

1 Richards, S. *et al.* Standards and guidelines for the interpretation of sequence variants: a joint consensus recommendation of the American College of Medical Genetics and Genomics and the Association for Molecular Pathology. Genet Med 17, 405-424 (2015).

2 Bramswig, N. C. *et al.* Heterozygosity for ARID2 loss-of-function mutations in individuals with a Coffin-Siris syndrome-like phenotype. Hum Genet 136, 297-305 (2017).

3 Shang, L. *et al.* Mutations in ARID2 are associated with intellectual disabilities. Neurogenetics 16, 307-314 (2015).

4 Wieczorek, D. *et al.* A comprehensive molecular study on Coffin-Siris and Nicolaides-Baraitser syndromes identifies a broad molecular and clinical spectrum converging on altered chromatin remodeling. Hum Mol Genet 22, 5121-5135 (2013).

5 Dobyns, W. B. & Das, S.PAFAH1B1-Associated Lissencephaly/Subcortical Band Heterotopia. in GeneReviews (eds M. P. Adam *et al.*) (1993)

6 Platzer, K. *et al.* GRIN2B encephalopathy: novel findings on phenotype, variant clustering, functional consequences and treatment aspects. J Med Genet 54, 460-470 (2017).

7 Lemke, J. R. *et al.* GRIN2B mutations in West syndrome and intellectual disability with focal epilepsy. Ann Neurol 75, 147-154 (2014).

8 Di Donato, N. *et al.* Analysis of 17 genes detects mutations in 81% of 811 patients with lissencephaly. Genet Med 20, 1354-1364 (2018).
